# Combining modelling and experimental approaches to explain how calcium signatures are decoded by calmodulin‐binding transcription activators (CAMTAs) to produce specific gene expression responses

**DOI:** 10.1111/nph.13428

**Published:** 2015-04-27

**Authors:** Junli Liu, Helen J. Whalley, Marc R. Knight

**Affiliations:** ^1^ School of Biological and Biomedical SciencesDurham Centre for Crop Improvement TechnologyDurham UniversitySouth RoadDurhamDH1 3LEUK; ^2^ Cell Signalling GroupCancer Research UK Manchester InstituteThe University of ManchesterManchesterM20 4BXUK

**Keywords:** Arabidopsis, calcium signatures, calmodulin (CaM), calmodulin‐binding transcription activators (CAMTAs), gene expression, mathematical modelling

## Abstract

- Experimental data show that *Arabidopsis thaliana* is able to decode different calcium signatures to produce specific gene expression responses. It is also known that calmodulin‐binding transcription activators (CAMTAs) have calmodulin (CaM)‐binding domains. Therefore, the gene expression responses regulated by CAMTAs respond to calcium signals. However, little is known about how different calcium signatures are decoded by CAMTAs to produce specific gene expression responses.
- A dynamic model of Ca^2+^–CaM–CAMTA binding and gene expression responses is developed following thermodynamic and kinetic principles. The model is parameterized using experimental data. Then it is used to analyse how different calcium signatures are decoded by CAMTAs to produce specific gene expression responses.
- Modelling analysis reveals that: calcium signals in the form of cytosolic calcium concentration elevations are nonlinearly amplified by binding of Ca^2+^, CaM and CAMTAs; amplification of Ca^2+^ signals enables calcium signatures to be decoded to give specific CAMTA‐regulated gene expression responses; gene expression responses to a calcium signature depend upon its history and accumulate all the information during the lifetime of the calcium signature.
- Information flow from calcium signatures to CAMTA‐regulated gene expression responses has been established by combining experimental data with mathematical modelling.

## Introduction

Plants are sessile organisms and therefore they must adapt their metabolism, growth and architecture to a changing environment. The majority of their defence against stress is realized by changes in gene expression in order to produce proteins required to combat the conditions they encounter. It is thus vital that the correct proteins are produced in response to different environmental conditions; that is, different genes need to be switched on in response to different stimuli. Calcium is a ubiquitous second messenger in eukaryotes and it is a ubiquitous intermediate between stimulus perception and responses in plants. It has been observed that different stimuli produce calcium signatures with different characteristics in plants (McAinsh *et al*., 1995; Allen *et al*., 2001; Love *et al*., 2004; Miwa *et al*., 2006; McAinsh & Pittman, 2009; Dodd *et al*., 2010; Kudla *et al*., 2010; Short *et al*., 2012). Given that calcium is an intermediate between stimulus perception and gene expression (Whalley *et al*., 2011), it is possible that the specific characteristics of the calcium signatures produced by different stresses encode stimulus‐specific information. Recent experimental data demonstrate that *Arabidopsis thaliana* is able to decode specific calcium signatures and interpret them, leading to distinct gene expression profiles (Whalley *et al*., 2011; Whalley & Knight, 2013).

Calmodulin‐binding transcription activators (CAMTAs) are well‐characterized Ca^2+^/calmodulin (CaM)‐regulated transcription factors (Kim *et al*., 2009; Galon *et al*., 2010; Reddy *et al*., 2011; Bickerton & Pittman, 2012; Poovaiah *et al*., 2013), and they have CaM‐binding domains (Finkler *et al*., 2007). Therefore, gene expression responses regulated by CAMTAs respond to calcium signals (Whalley *et al*., 2011; Whalley & Knight, 2013).

Although experimental data (Whalley *et al*., 2011; Whalley & Knight, 2013) show that *A. thaliana* is able to decode different calcium signatures to produce specific gene expression responses, little is known about how complex calcium signatures are decoded to generate gene expression responses. In this work, we establish the principles of information flow from calcium signatures to CAMTA‐regulated gene expression responses by combining experimental data with mathematical modelling.

## Materials and Methods

### A dynamic model describing the information flow from calcium signatures to CAMTA‐regulated gene expression

**Figure 1:**
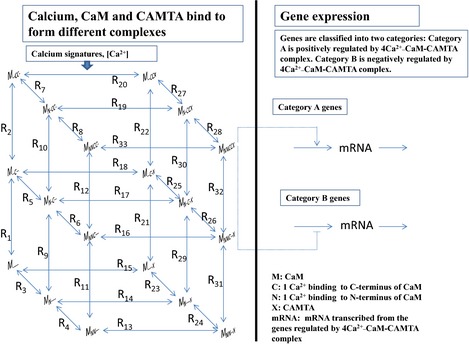
A dynamic model that describes the information flow from calcium signatures to calmodulin‐binding transcription activator (CAMTA)‐regulated gene expression in *Arabidopsis thaliana*. Left panel: Ca^2+^, calmodulin (CaM) and CAMTA bind to form different complexes. When [Ca^2+^] changes, this binding process responds following thermodynamic principles. Right panel: gene expression is regulated by the active complex 4Ca^2+^–CaM–CAMTA (MNNCCX) using the two simplest gene expression mechanisms. This figure illustrates a generic model for studying the information flow from calcium signatures to CAMTA‐regulated gene expression.

The information flow from calcium signatures to CAMTA‐regulated gene expression in *Arabidopsis thaliana* is described in Fig. 1. The left pane of Fig. 1 describes the binding of Ca^2+^, CaM and CAMTAs. CaM has two pairs of Ca^2+^‐binding EF‐hand domains located at the N and C termini, respectively (Finn & Forsen, 1995; Valeyev *et al*., 2008). Ca^2+^‐binding kinetics for the pairs of EF‐hands at the N and C termini are significantly different and their Ca^2+^‐binding kinetics display cooperativity (Linse *et al*., 1991). The cooperative binding between Ca^2+^ and the four binding sites of CaM has been previously subjected to both experimental and modelling studies (Fajmut *et al*., 2005; Shifman *et al*., 2006; Pepke *et al*., 2010) and the kinetic parameters have been determined (Shifman *et al*., 2006; Pepke *et al*., 2010). Table 1 summarizes those parameters (Shifman *et al*., 2006; Pepke *et al*., 2010). In addition, experimental data show that the CAMTA proteins consist of multiple functional domains associated with binding of CaM and CaM‐like proteins (Bouche *et al*., 2002; Finkler *et al*., 2007). Mapping of a Ca^2+^‐dependent CaM‐binding domain in *A. thaliana* AtCAMTA1 revealed a single high‐affinity binding site (the binding affinity *K*_d_ = 1.2 × 10^−3^ μM) (Bouche *et al*., 2002; Finkler *et al*., 2007) and a similar binding site exists in rice (*Oryza sativa*) (Choi *et al*., 2005). As binding of the Ca^2+^–CaM complex to CAMTAs is tighter than binding of free CaM to CAMTAs (Bouche *et al*., 2002; Finkler *et al*., 2007), the *K*_d_ for R15 in Fig. 1 is always larger than 1.2 × 10^−3^ μM. These parameters are also included in Table 1.

**Table 1:** Parameters for the model described in Fig. 1

1. Parameters derived using experimental data for the binding of Ca^2+^, CaM and CAMTA (left pane of Fig. 1)			
Reaction	Reaction description	Equilibrium constant (*K*_d_)	Kinetic constants (*k*_on_; *k*_off_)
R1, R9, R11	Binding of first Ca^2+^ to CaM C terminus	10 μM (Linse *et al*., 1991; Shifman *et al*., 2006; Kubota *et al*., 2007; Pepke *et al*., 2010)	*k*_on_ = 4 μM^−1^ s^−1^; *k*_off_ = 40 s^−1^ (Martin *et al*., 1992; Persechini *et al*., 1996; Gaertner *et al*., 2004; Pepke *et al*., 2010)
R2, R10, R12	Binding of second Ca^2+^ to CaM C terminus	0.925 μM (Linse *et al*., 1991; Shifman *et al*., 2006; Kubota *et al*., 2007; Pepke *et al*., 2010)	*k*_on_ = 10 μM^−1^ s^−1^; *k*_off_ = 9.25 s^−1^ (Gaertner *et al*., 2004; Pepke *et al*., 2010)
R3, R5, R7	Binding of first Ca^2+^ to CaM N terminus	25 μM (Linse *et al*., 1991; Shifman *et al*., 2006; Kubota *et al*., 2007; Pepke *et al*., 2010)	*k*_on_ = 100 μM^−1^ s^−1^; *k*_off_ = 2500 s^−1^ (Brown *et al*., 1997; Peersen *et al*., 1997; Gaertner *et al*., 2004; Pepke *et al*., 2010)
R4, R6, R8	Binding of second Ca^2+^ to CaM N terminus	5 μM (Linse *et al*., 1991; Shifman *et al*., 2006; Kubota *et al*., 2007; Pepke *et al*., 2010)	*k*_on_ = 150 μM^−1^ s^−1^; *k*_off_ = 750 s^−1^ (Brown *et al*., 1997; Peersen *et al*., 1997; Gaertner *et al*., 2004; Pepke *et al*., 2010)
R14	Binding of Ca^2+^–CaM complex to CAMTA	1.2 × 10^−3^ μM (Bouche *et al*., 2002; Finkler *et al*., 2007)	*k*_on_ = 1 μM^−1^ s^−1^; *k*_off_ = 1.2 × 10^−3^ s^−1^ Notes: *k*_on_ is an adjustable parameter in this work. *k*_off_ = *K*_d_ *k*_on_
R15	Binding of free CaM to CAMTA	*K*_d(R14)_/*P* = 1.2 × 10^−3^ μM/*P*. *P* = 0.1, which is always smaller than 1, is an adjustable parameter, indicating that binding of Ca^2+^–CaM complex to CAMTA is tighter than binding of free CaM to CAMTA (Bouche *et al*., 2002; Finkler *et al*., 2007)	*k*_on_ = *k*_on(R14)_/*Q*. *Q* = 1.0 is an adjustable parameter and it describes the cooperative binding between CaM and CAMTA in the presence of Ca^2+^ due to on binding rate. *k*_off_ = *K*_d_ *k*_on_ = (*K*_d(R14)_ *k*_on(R14)_)/(PQ) = *k*_off(R14)_/(PQ)
2. Parameters derived based on the detailed balance conditions following thermodynamic principles and the assumption that the affinity for the binding of any Ca^2+^–CaM complex to CAMTA is always the same (left pane of Fig. 1)			
*K*_d(R13)_ = *K*_d(R14)_ = *k*_d(R16)_ = *k*_d(R17)_ = *K*_d(R18)_ = *K*_d(R19)_ = *K*_d(R20)_ = *K*_d(R33)_, *K*_d(R2)_ = *K*_d(R22)_, *K*_d(R4)_ = *K*_d(R24)_, *K*_d(R5)_ = *K*_d(R25)_, *K*_d(R6)_ = *K*_d(R26)_, *K*_d(R7)_ = *K*_d(R27)_, *K*_d(R8)_ = *K*_d(R28)_, *K*_d(R9)_ = *K*_d(R29)_, *K*_d(R10)_ = *K*_d(R30)_, *K*_d(R11)_ = *K*_d(R31)_, *K*_d(R12)_ = *K*_d(R32)_ As long as the binding affinities (*K*_d_) for two reactions are the same, we consider their respective *k*_on_ and *k*_off_ are also the same.			
3. Parameters for gene expression (right pane of Fig. 1)			
*k*_1_ = 5.0 × 10^−6^ μM s^−1^, *k*_2_ = 5.0 × 10^−2^ μM s^−1^, *n* = 2, *k*_3_ = 3.75 × 10^−4^ s^−1^, *k*_4_ = 1.1 × 10^−2^ μM			

CaM, calmodulin; CAMTA, calmodulin‐binding transcription activator.

The unknown equilibrium constants and on/off rates in the left pane of Fig. 1 are derived using the detailed balance condition following thermodynamic principles. For example, following the detailed balance condition, Eqn (1) is always valid.

(1) $$K_{d(R15)}K_{d(R23)}=K_{d(R3)}K_{d(R14)}$$

Eqn (1) leads to Eqn (2).

(2) $$K_{d(R23)}=\frac{K_{d(R3)}K_{d(R14)}}{K_{d(R15)}}=K_{d(R3)}P$$

As binding of the Ca^2+^–CaM complex to CAMTAs is tighter than binding of free CaM to CAMTAs (Bouche *et al*., 2002; Finkler *et al*., 2007), *P* is always < 1 (Table 1). *P* describes the cooperative binding between CaM and CAMTAs in the presence of Ca^2+^. As *P* has not been experimentally determined, it is an adjustable parameter in this work. Moreover, as *K*_d_ is *K*_off_/*K*_on_, the difference between *K*_d(R3)_ and *K*_d(R23)_ or the difference between *K*_d(R14)_ and *K*_d(R15)_ could be caused by the difference in *k*_on_, *k*_off_ or both. In order to examine the effects of changes in *k*_on_, *k*_off_ or both on modelling results, we define

(3) $$k_{\mathrm{on}(R15)}=k_{\mathrm{on}(R14)}/Q.$$

where *Q* is an adjustable parameter. If *Q* = 1.0, this implies that cooperativity is realized solely by the changes in *k*_off_. Similarly, *Q* = 1/*P* implies that cooperativity is realized solely by the changes in *k*_on_. Other values of *Q* imply that cooperativity is realized by the changes in both *k*_on_ and *k*_off_. After applying the detailed balance condition following thermodynamic principles to all other loops in Fig. 1, the equilibrium constants (*K*_d_) are linked.

Based on experimental data, binding of the Ca^2+^–CaM complex to CAMTAs is tighter than binding of free CaM to CAMTAs (Bouche *et al*., 2002; Finkler *et al*., 2007). However, experimental measurements are not able to identify the binding affinity of each different Ca^2+^–CaM complex (i.e. with different numbers of calcium ions at different positions) to CAMTAs. Here, we assume that the affinity for the binding of any Ca^2+^–CaM complex to CAMTAs is always the same, regardless of how many Ca^2+^ bind to CaM. The advantage of this assumption is to greatly reduce adjustable parameters. However, for the sake of completeness, we have randomly tested the effects of different binding affinities for the binding between some Ca^2+^–CaM complexes and CAMTAs. Under the conditions that binding of the Ca^2+^–CaM complex to CAMTAs is tighter than binding of free CaM to CAMTAs (Bouche *et al*., 2002; Finkler *et al*., 2007), we have tested the effects of changes in binding affinity of up to 2 orders with reference to a value of 1.2 × 10^−3^ μM for some Ca^2+^–CaM complexes (e.g. R20 and R33). The qualitative conclusions we will draw in this work do not change if these binding affinities change, as shown in Supporting Information Figs S1–S4. After introducing the detailed balance condition following thermodynamic principles and based on the assumption that the affinity for the binding of any Ca^2+^–CaM complex to CAMTAs is always the same, we are able to derive all other unknown equilibrium constants and on/off rates, as summarized in Table 1.

After using the parameters determined experimentally and introducing thermodynamic constraints, there are only five adjustable parameters left for the left pane of Fig. 1, as summarized as follows. *P* describes the cooperative binding between CaM and CAMTAs in the presence of Ca^2+^; *k*_on(R14)_ is the on rate for the binding of the Ca^2+^–CaM complex to CAMTAs; *Q* describes how the cooperative binding between CaM and CAMTAs in the presence of Ca^2+^ is realized by *k*_on_, *k*_off_ or both. CaM_t describes the total concentration of CaM, which is the summation of free CaM and all CaM complexes. X_t describes the total concentration of CAMTAs, which is the summation of free CAMTAs and all CAMTA complexes.

The mass balance of each complex in the left pane of Fig. 1 is described using a differential equation (Notes S1). By coupling these differential equations together, we are able to calculate the concentration of any complex for any calcium signature at any time. It is known that 4Ca^2+^–CaM is the active CaM–Ca^2+^ binding complex (Pifl *et al*., 1984). Therefore, this work assumes that the complex 4Ca^2+^–CaM–CAMTA (MNNCCX in Fig. 1) is the active complex for the gene expression response.

The right pane of Fig. 1 describes CAMTA‐regulated gene expression. CAMTAs can be either activators or suppressors of gene expression, as evidenced by the experiments using CAMTA mutants (Galon *et al*., 2008; Doherty *et al*., 2009). In addition, the process of gene expression may have multiple entry points of Ca^2+^ signal as a consequence of the interactions between Ca^2+^ signal and CAMTAs (Miller *et al*., 2013; Zhang *et al*., 2014). Clearly, different genes may be regulated by different expression mechanisms. Even if they are regulated by the same mechanism, the parameter values controlling their expression may be different. Moreover, for most genes, gene expression mechanisms have not been experimentally determined. In this work, our focus is to investigate how different calcium signatures are decoded by CAMTAs to produce specific gene expression responses. Our primary interest is to establish the information flow from calcium signatures to gene expression rather than the gene expression mechanisms themselves. Therefore, here we use as simple as possible generic gene expression mechanisms. Our method can be generally extended to include any gene expression mechanism by replacing the right pane of Fig. 1, if the specific expression mechanism of that specific gene is known.

The simplest gene expression process includes: (1) gene transcription is activated or supressed by a transcription factor; and (2) the mRNA is decayed or consumed. The right pane of Fig. 1 describes these simplest mechanisms. For category A genes in Fig. 1, the differential equation for describing gene expression is as follows.

(4) $$\frac{d\left[\text{mRNA}\right]}{\text{at}}=k_{1}+\frac{k_{2}{\left(\frac{\left[\text{MNNCCX}\right]}{k_{4}}\right)}^{n}}{1+{\left(\frac{\left[\text{MNNCCX}\right]}{k_{4}}\right)}^{n}}-k_{3}\left[\text{mRNA}\right]$$

(*k*_1_, the base rate for gene transcription; *k*_2_, the maximal rate for CAMTA‐regulated gene transcription; *k*_3_, the decay rate constant for the mRNA; *k*_4_, the binding affinity between the 4Ca^2+^–CaM–CAMTA complex and DNA; *n*, the Hill coefficient.)

Similarly, for category B genes in Fig. 1, the differential equation for describing gene expression is as follows.

(5) $$\frac{\text{d[mRNA]}}{\text{at}}=k_{1}+\frac{k_{2}}{1+{\left(\frac{\left[\text{MNNCCX}\right]}{k_{4}}\right)}^{n}}-k_{3}\left[\text{mRNA}\right]$$

The parameters for gene transcription are included in Table 1. In the Results section, we will also test and discuss how the parameters relating to gene transcription affect the information flow from calcium signatures to gene expression.

### Time delay

It is evident that the information flow from calcium signatures to changes in gene expression will generally be subjected to a time delay. When a calcium signal emerges, a change in gene expression cannot occur instantly, as the transcriptional pre‐initiation complex (containing specific transcription factors, e.g. CAMTAs, general transcription factors, mediators and RNA polymerase) needs to be recruited and assembled and an elongation complex needs to form to allow transcription of the coding region (Lee & Young, 2000). In this work, we consider that there is a time delay, *τ*, between the calcium signal and the gene expression response. After all the concentrations in the left pane of Fig. 1 are calculated, the complex 4Ca^2+^–CaM–CAMTA induces gene expression after a time delay (*τ*). The effects of *τ* on modelling results will be examined.

### Numerical method

The model is implemented using the simulator Berkeley Madonna (www.berkeleymadonna.com). The Rosenbrock (Stiff) method is used with a tolerance of 1.0 × 10^−5^. Much smaller tolerances have also been tested and the numerical results show that further reduction of tolerances does not improve the accuracy of numerical results. Before a calcium signature is introduced, the system of ordinary differential equations is settled at a steady state using the average Ca^2+^ concentration of the control experiment as an input. When a calcium signature is introduced, the response of the system of ordinary differential equations is calculated using the time‐dependent Ca^2+^ concentration as an input.

## Results

### Ca^2+^ signals are nonlinearly amplified as a result of the Ca^2+^–CaM–CAMTA interaction

**Figure 2:**
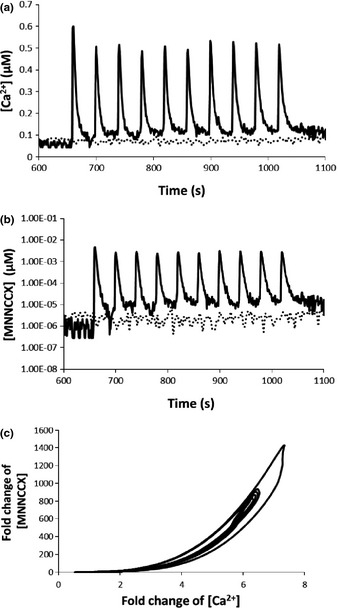
Oscillatory calcium signature induced in experiments that use controlled electrical stimulations (Whalley *et al*., 2011) and amplification of Ca^2+^ signal as a result of 4Ca^2+^–CaM–CAMTA binding in *Arabidopsis thaliana*. (a) Solid line: experimental Ca^2+^ elevation. Dashed line: control. (b) Computational results for the response of the active complex 4Ca^2+^–CaM–CAMTA to the calcium signature (solid line) and to the control experiment (dashed line), respectively. (c). Fold‐change analysis shows that Ca^2+^ signals are nonlinearly amplified by 4Ca^2+^–CaM–CAMTA binding. CAMTA, calmodulin‐binding transcription activator; CaM, calmodulin.

**Figure 3:**
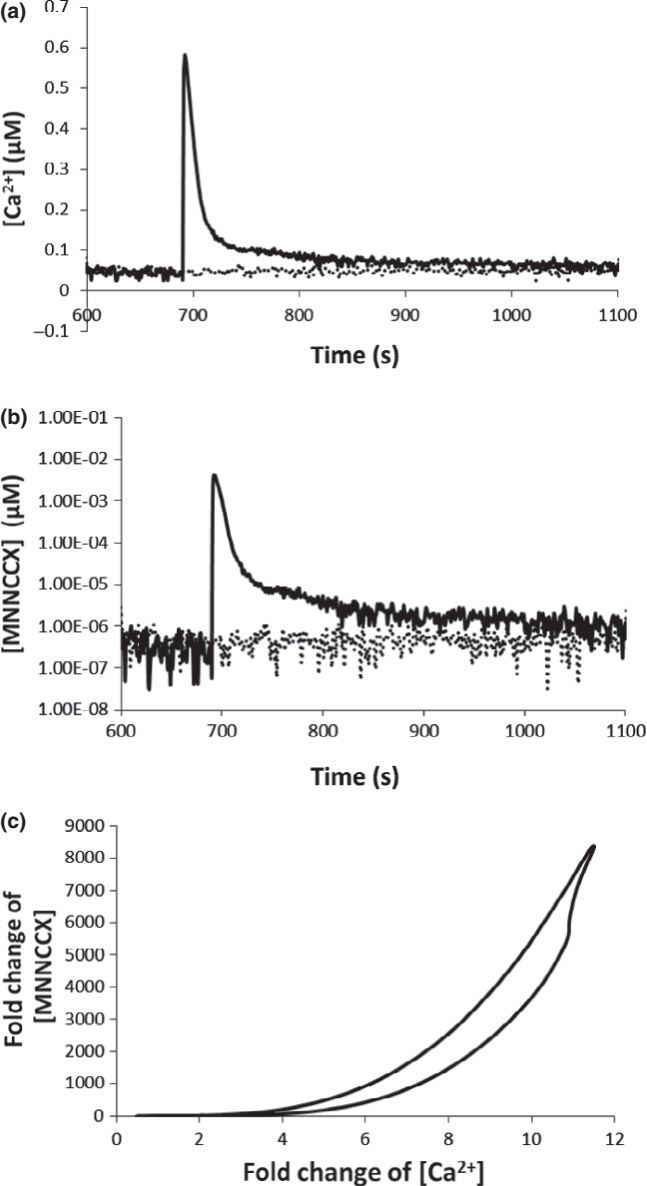
Transient calcium signature induced in experiments that use controlled electrical stimulations (Whalley *et al*., 2011) and amplification of Ca^2+^ signal as a result of 4Ca^2+^–CaM–CAMTA binding in *Arabidopsis thaliana*. (a) Solid line: experimental Ca^2+^ elevation. Dashed line: control. (b) Computational results for response of the active complex 4Ca^2+^–CaM–CAMTA to the calcium signature (solid line) and to the control experiment (dashed line), respectively. (c) Fold‐change analysis shows that Ca^2+^ signals are nonlinearly amplified by 4Ca^2+^–CaM–CAMTA binding. CAMTA, calmodulin‐binding transcription activator; CaM, calmodulin.

**Figure 4:**
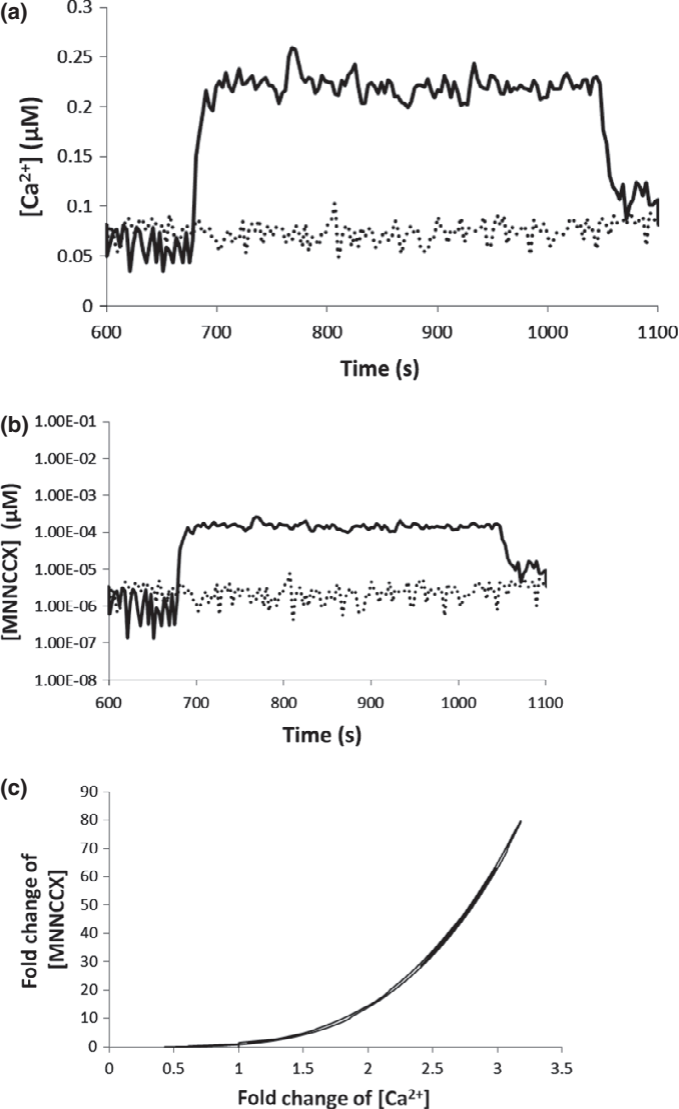
Prolonged calcium signature induced in experiments that use controlled electrical stimulations (Whalley *et al*., 2011) and amplification of Ca^2+^ signal as a result of 4Ca^2+^–CaM–CAMTA binding in *Arabidopsis thaliana*. (a) Solid line: experimental Ca^2+^ elevation. Dashed line: control. (b) Computational results for response of the active complex 4Ca^2+^–CaM–CAMTA to the calcium signature (solid line) and to the control experiment (dashed line), respectively. (c) Fold‐change analysis shows that Ca^2+^ signals are nonlinearly amplified by 4Ca^2+^–CaM–CAMTA binding. CAMTA, calmodulin‐binding transcription activator; CaM, calmodulin.

As shown in Fig. 1, under thermodynamic constraints, CaM binds to Ca^2+^, forming complexes with different numbers of calcium ions at different positions. It is known that 4Ca^2+^–CaM is the active CaM–Ca^2+^ binding complex (Pifl *et al*., 1984). Figs 2, 3 and 4 summarize the amplification of Ca^2+^ signals for three different Ca^2+^ signatures, which were experimentally generated (Whalley *et al*., 2011). As a result of the interaction of Ca^2+^–CaM–CAMTA, Ca^2+^ signals are nonlinearly amplified into the signals of the active functioning complex 4Ca^2+^–CaM–CAMTA.

The three Ca^2+^ signatures (Figs 2a, 3a, 4a) are the inputs for the interaction of Ca^2+^–CaM–CAMTA (Fig. 1), and the respective concentrations of the active functioning complex 4Ca^2+^–CaM–CAMTA for the three Ca^2+^ signatures are shown in Figs 2(b), 3(b), and 4(b). Fig. 2(c) shows that, for the oscillatory Ca^2+^ signature (Fig. 2a), a *c*. 7‐fold change in Ca^2+^ concentration (relative to the experimental measurement of average Ca^2+^ concentration in control experiments) is amplified to a *c*. 1400‐fold change in the concentration of the 4Ca^2+^–CaM–CAMTA complex (relative to the average computed concentration using Ca^2+^ concentration in control experiments). Fig. 3(c) shows that, for the transient Ca^2+^ signature (Fig. 3a), a *c*. 12‐fold change in Ca^2+^ concentration is amplified to a *c*. 8000‐fold change in the concentration of the 4Ca^2+^–CaM–CAMTA complex. Similarly, Fig. 4(c) shows that, for the prolonged Ca^2+^ signature (Fig. 4a), a *c*. 3.2 ‐fold change in Ca^2+^ concentration is amplified to a *c*. 80‐fold change in the concentration of the 4Ca^2+^–CaM–CAMTA complex.

Combination of Figs 2, 3 and 4 reveals that the amplification of Ca^2+^ signals by the interaction of Ca^2+^–CaM–CAMTA is nonlinear. For example, with reference to the steady‐state value of the Ca^2+^ concentration and the corresponding concentration of the 4Ca^2+^–CaM–CAMTA complex, a *c*. 2‐, 4‐ or 10‐fold increase in Ca^2+^ concentration leads to a *c*. 10‐, 200‐ or 5000‐fold increase in 4Ca^2+^–CaM–CAMTA, respectively. Therefore, although the fold changes of Ca^2+^ concentration in three Ca^2+^ signatures are relatively small, the resulting fold changes of the active complex (4Ca^2+^–CaM–CAMTA) are large and different for each individual signature.

**Figure 5:**
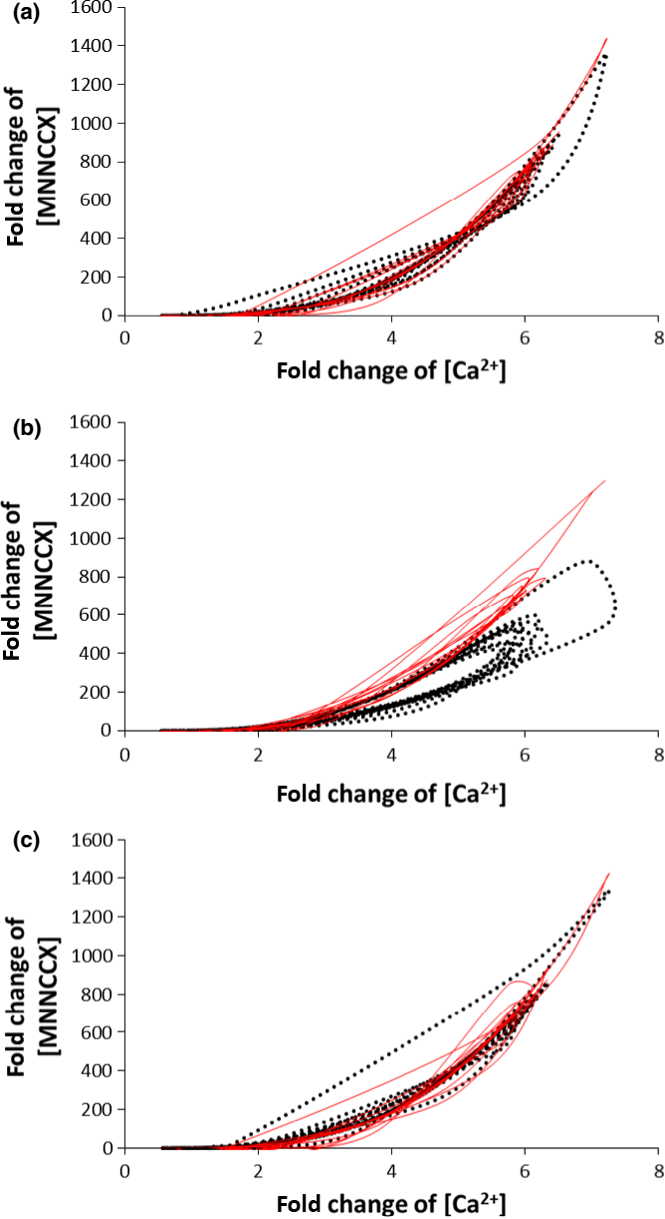
Evaluating the effects of the adjustable parameters on the amplification of Ca^2+^ signals. (a) Effects of altering the on rate for the binding between the Ca^2+^–calmodulin (CaM) complex and calmodulin‐binding transcription activators (CAMTAs) (*k*_on(R14)_) on the amplification of Ca^2+^ signals. Solid line: *k*_on(R14)_ = 100 μM^−1^ s^−1^. Dashed line: *k*_on(R14)_ = 0.01 μM^−1^ s^−1^. The reference value is *k*_on(R14)_ = 1 μM^−1^ s^−1^ (Fig. 2). (b) Effects of altering the cooperative binding between CaM and CAMTA in the presence of Ca^2+^ by altering the binding rate (*Q* in Eqn (3)) on the amplification of Ca^2+^ signals. Solid line: *Q* = 0.01 μM^−1^ s^−1^. Dashed line: *Q* = 100 μM^−1^ s^−1^. The reference value is *Q* = 1 μM^−1^ s^−1^ (Fig. 2). (c) Effects of altering the total CAMTA concentration on the amplification of Ca^2+^ signals. Solid line: X_t = 1000 μM. Dashed line: X_t = 0.1 μM. The reference value is 10 μM (Fig. 2).

We have further examined how the five adjustable parameters relating to the left pane of Fig. 1 affect the modelling results shown in Figs 2, 3, 4. The nonlinear fold‐change relationship between Ca^2+^ signals and the corresponding active complex (4Ca^2+^–CaM–CAMTA) always exists across a wide range of values for these five adjustable parameters, as shown in Figs 5, S5 and S6.

Fig. 5(a) shows the effects of varying the on rate for the binding between the Ca^2+^–CaM complex and CAMTAs (*k*_on(R14)_) on the amplification of Ca^2+^ signals. For a 4‐order change in *k*_on(R14)_, the amplification of Ca^2+^ signals is qualitatively similar. If *k*_on(R14)_ is further increased from 100 μM^−1^ s^−1^, the amplification of Ca^2+^ signals is similar to the solid line in Fig. 5(a). However, if *k*_on(R14)_ is further decreased from 0.01 μM^−1^ s^−1^, the amplification of Ca^2+^ signals will be markedly smaller. Fig. 5(b) shows that effects of varying the cooperative binding between CaM and CAMTAs in the presence of Ca^2+^ by altering the binding rate (*Q* in Eqn (3)). For a 4‐order change, the qualitative trend for the amplification of Ca^2+^ signals is always similar. In addition, we have tested the effects of varying the cooperative binding between CaM and CAMTAs in the presence of Ca^2+^ (P in Eqn (2)) in a 3‐order range, as P can only be increased to 1.0. Fig. S5 shows that the qualitative trend for the amplification of Ca^2+^ signals is always similar. Fig. 5(c) shows the effects of varying the total CAMTA concentration on the amplification of Ca^2+^ signals. Change of the total CAMTA concentration in a 4‐order range gives rise to qualitatively similar amplification of Ca^2+^ signals. In a similar manner, Fig. S6 shows that the qualitative trend for the amplification of Ca^2+^ signals is always similar for a 4‐order change of the total CaM concentration. In addition, we have also examined the effects of simultaneous variations of all five adjustable parameters. When all parameters are varied, there are a large number of possible combinations. In this work, therefore, we are only able to test certain combinations. As shown in Figs S7 and S8, the qualitative trend for the amplification of Ca^2+^ signatures is similar when all parameters vary.

Therefore, as a result of the interaction of Ca^2+^–CaM–CAMTA, Ca^2+^ signals are always nonlinearly amplified (Figs 2c, 3c, 4c). Moreover, for three different calcium signatures (Figs 2a, 3a, 4a), as a result of the differences in the amplitude of Ca^2+^ signatures, the maximum amplification fold change of the three calcium signatures is significantly different (Figs 2, 3, 4).

### Amplification of Ca^2+^ signals enables calcium signatures to be decoded to give specific CAMTA‐regulated gene expression responses

Experimental data for fold change in CAMTA‐regulated gene expression level for three calcium signatures (Figs 2a, 3a, 4a) are included in Table 2. We defined CAMTA‐regulated genes as the 20 genes that were induced by any calcium signature described in Whalley *et al*. (2011) which contained the CAMTA‐binding motif 5′‐ACGCGT‐3′ within 500 bp of their promoters (Whalley *et al*., 2011). As shown in Table 2, both oscillatory (Fig. 2a) and transient (Fig. 3a) calcium signatures are able to induce > 1.5‐fold expression change in CAMTA‐regulated genes, while the prolonged (Fig. 4a) calcium signature cannot induce > 1.5‐fold change in any CAMTA‐regulated gene (Whalley *et al*., 2011). As the elevated Ca^2+^ increases CAMTA‐regulated gene expression for oscillatory (Fig. 2a) and transient (Fig. 3a) calcium signatures, we consider that, under our experimental conditions (Whalley *et al*., 2011), Ca^2+^ only activates (but does not decrease) CAMTA‐regulated gene expression (Table 2). Thus, we use Eqn (4) to calculate the effects of different calcium signatures on CAMTA‐regulated gene expression.

**Table 2:** Experimental results for the fold change of calmodulin‐binding transcription activator (CAMTA)‐regulated gene expression at 1 h in *Arabidopsis thaliana* for the three calcium signatures that were induced using controlled electrical stimulations (Whalley *et al*., 2011)

Arabidopsis Genome Initiative (AGI) code	Fold change for oscillatory calcium signature; Fig. 2a	Fold change for transient calcium signature; Fig. 3a	Fold change for prolonged calcium signature; Fig. 4a
AT2G20630	3.13	2.36	Not induced
AT3G10300	Not induced	2.14	Not induced
AT3G18420	1.71	2.06	Not induced
AT1G19180	1.54	2.26	Not induced
AT5G15650	1.80	2.27	Not induced
AT3G05500	3.14	3.90	Not induced
AT1G07890	1.58	2.12	Not induced
AT1G18610	Not induced	4.56	Not induced
AT1G19380	1.89	2.29	Not induced
AT1G63750	3.08	No data	Not induced
AT3G03020	1.82	2.11	Not induced
AT3G19150	2.20	1.85	Not induced
AT3G43680	Not induced	5.49	Not induced
AT3G45970	Not induced	2.02	Not induced
AT4G19200	2.18	2.02	Not induced
AT4G22610	1.62	1.99	Not induced
AT4G29670	2.26	1.89	Not induced
AT4G30210	1.74	2.53	Not induced
AT5G24810	2.11	2.06	Not induced
AT5G45350	2.40	3.24	Not induced

‘Not induced’ refers to a < 1.5‐fold change (Whalley *et al*., 2011).

**Figure 6:**
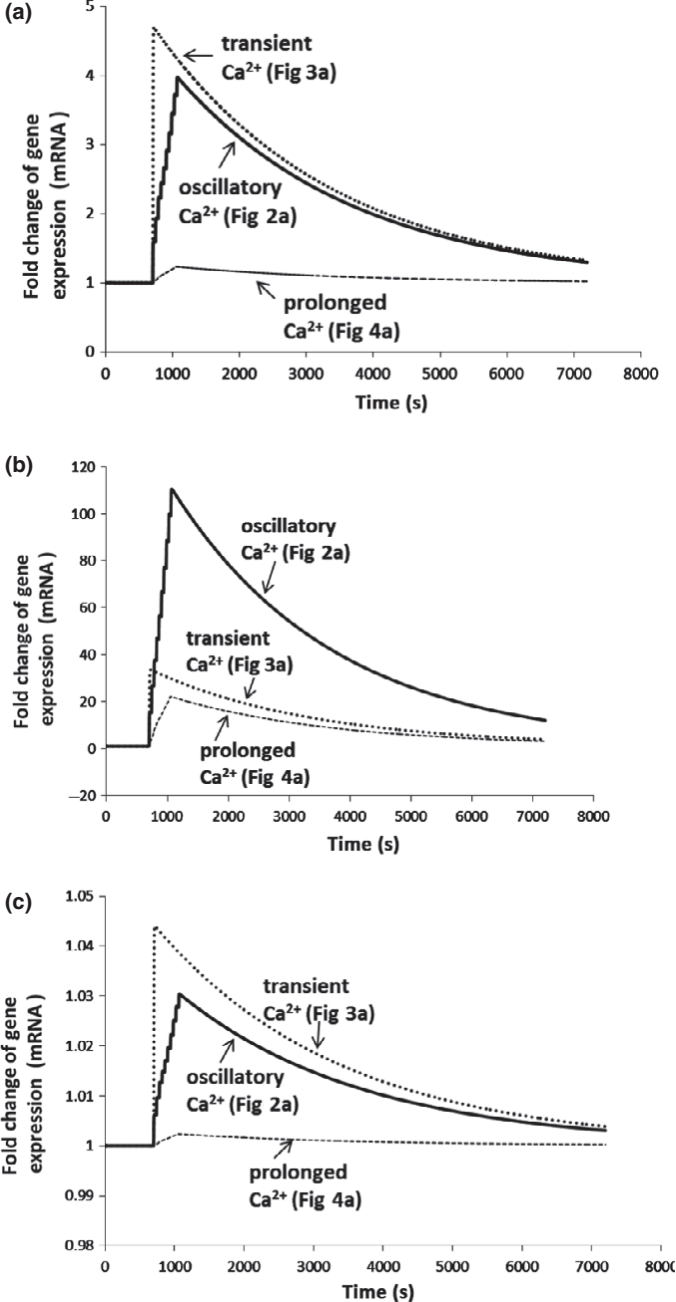
Fold change in gene expression induced by three different calcium signatures with three binding affinities between the active complex 4Ca^2+^–CaM–CAMTA and DNA in *Arabidopsis thaliana*. The delay time between calcium signature and gene expression is 600 s for all three calcium signatures. (a) Binding affinity (*K*_d_) is 1.1 × 10^−2^ μM. Both oscillatory and transient calcium signatures induce *c*. 2‐fold gene expression increase at 1 h, while the prolonged calcium signature induces *c*. 1.05‐fold gene expression increase at 1 h. (b) Binding affinity (*K*_d_) is 1.1 × 10^−3^ μM. Oscillatory, transient and prolonged calcium signatures induce *c*. 43‐, 12‐ and 9‐fold gene expression increases at 1 h, respectively. (c) Binding affinity (*K*_d_) is 1.1 × 10^−1^ μM. Oscillatory, transient and prolonged calcium signatures all induce < 1.02‐fold gene expression increases at 1 h. CAMTA, calmodulin‐binding transcription activator; CaM, calmodulin.

The capability of the Ca^2+^–CaM–CAMTA interaction to nonlinearly amplify Ca^2+^ signals allows different calcium signatures to be differentially decoded to generate specific gene expression responses. If Ca^2+^ signals were not amplified by the Ca^2+^–CaM–CAMTA interaction, the fold changes in the three calcium signatures (Figs 2a, 3a, 4a) would be small (from *c*. 3.5‐fold (for a prolonged calcium signature; Fig. 4a) to *c*. 11‐fold (for a transient calcium signature; Fig. 3a). Such differences in Ca^2+^ signals would, on their own, be too small to be distinguished and to allow different gene expression responses if they were not amplified. Thus, the role of the Ca^2+^–CaM–CAMTA interaction in amplifying Ca^2+^ signals is important for inducing specific gene expression responses. Fig. 6 shows how different calcium signatures are decoded to generate specific gene expression responses by virtue of the Ca^2+^–CaM–CAMTA interaction.

As shown in Fig. 6(a), as a result of the large fold amplification of Ca^2+^ signals in oscillatory and transient calcium signatures (Figs 2a, 3a), large fold changes in gene expression level are induced. Similarly, as a result of the small fold amplification of Ca^2+^ signals in prolonged calcium signatures (Fig. 4a), only a small fold change in gene expression level is induced. Thus, at 1 h, the fold change of gene expression level is generally larger than 1.5‐fold (Fig. 6a) for oscillatory and transient calcium signatures (Figs 2a, 3a), while it is < 1.5‐fold (Fig. 6a) for the prolonged calcium signature (Fig. 4a). Figs S9, S10 and S11 show that fold change for all three calcium signatures at a specific time (e.g. 1 h) depends on the delay time, which is the time when gene expression starts to responds to Ca^2+^ signals. In Fig. 6(a), we assume that the delay time is always 600 s for three calcium signatures. If we assume that the delay time is different for different genes and/or different calcium signatures, the fold change of gene expression for oscillatory and transient calcium signatures at 1 h will change (Figs S9, S10). However, the fold change of gene expression for a prolonged calcium signature is always < 1.5‐fold, independently of the delay time (Fig. S11). Thus, Fig. 6(a) explains the experimental observations in Table 2 (Whalley *et al*., 2011), and it shows that calcium signatures are differentially decoded to give specific CAMTA‐regulated gene expression responses.

Modelling analysis further reveals that the binding affinity between the 4Ca^2+^–CaM–CAMTA complex and DNA is an important parameter for inducing gene expression by calcium signatures. To our knowledge, this parameter has not been experimentally determined. When the binding affinity is reduced to 1.1 × 10^−3^ μM from 1.1 × 10^−2^ μM, all three calcium signatures in Figs 2(a), 3(a) and 4(a) are able to induce different large fold changes in gene expression (Fig. 6b). At 1 h, oscillatory (Fig. 2a), transient (Fig. 3a) and prolonged (Fig. 4a) calcium signatures induce *c*. 43‐, 12‐ and 9‐fold changes in gene expression, respectively. Thus, Fig. 6(b) shows that even a relatively small fold amplification of calcium signals (e.g. a prolonged calcium signature; Fig. 4a) is able to induce a relatively large fold induction of gene expression, with an oscillatory calcium signature inducing the largest fold change in gene expression . By contrast, the largest fold change in gene expression is induced by a transient calcium signature (Fig. 3a) in Fig. 6(a). Comparison of Fig. 6(a,b) shows that the binding affinity between the 4Ca^2+^–CaM–CAMTA complex and DNA can change how CAMTA‐regulated gene expression responds to different calcium signatures. In addition, when the binding affinity is decreased to 1.1 × 10^−1^ μM from 1.1 × 10^−2^ μM, all three calcium signatures in Figs 2(a), 3(a) and 4(a) are unable to induce fold changes larger than 1.05 in gene expression (Fig. 6c). Therefore, gene expression induced by different calcium signatures is quantitatively dependent on the binding affinity between the 4Ca^2+^–CaM–CAMTA complex and DNA.

In addition, the effects of other parameters relating to gene expression were also examined. Increasing or decreasing *k*_1_ (the base rate for gene transcription) or *k*_2_ (the maximal rate for 4Ca^2+^–CaM–CAMTA complex‐regulated gene transcription) by 2‐fold does not qualitatively change the modelling results (Figs S12, S13). The Hill coefficient (*n*) taking a value of 1, 2 or 3 also qualitatively leads to similar results (Fig. S14). However, the decay constant of mRNA (*k*_3_) is an important parameter that affects the shape of the curve for gene expression (Fig. 6). If *k*_3_ is very small, gene expression continues to increases for the computational time we have tested (2 h). If *k*_3_ is very large, gene expression approaches the original steady state very quickly. An approximately 2‐fold increase or decrease of *k*_3_ from its reference value (3.75 × 10^−4^ s^−1^) generally maintains the shape of the curve for gene expression, as shown in Fig. 6 (Fig. S15).

### Gene expression response to a calcium signature depends on its history during its lifetime

**Figure 7:**
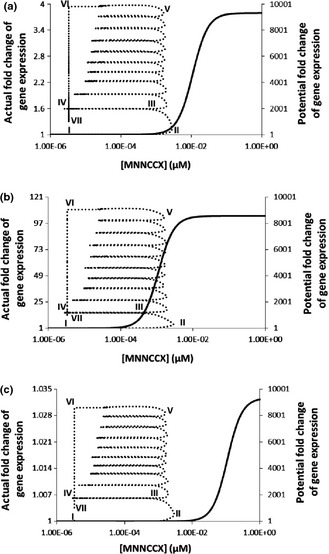
Gene expression accumulates all information during the lifetime of the oscillatory calcium signature (Fig. 2a) for three binding affinities between the active complex 4Ca^2+^–CaM–CAMTA and DNA in *Arabidopsis thaliana*. Solid line (right *y*‐axis): potential fold change of gene expression if the concentration of 4Ca^2+^–CaM–CAMTA stays at each concentration sufficiently long that a steady‐state is established at each concentration. Dashed line (left *y*‐axis): actual fold change of gene expression for 10 cycles of Ca^2+^ oscillation (Fig. 2a). Binding affinity (*K*_d_) between the active complex 4Ca^2+^–CaM–CAMTA and DNA: (a) 1.1 × 10^−2^ μM; (b) 1.1 × 10^−3^ μM; (c) 1.1 × 10^−1^ μM. CAMTA, calmodulin‐binding transcription activator; CaM, calmodulin.

Modelling analysis reveals that the gene expression response to a specific calcium signature depends on its history during its lifetime. Fig. 7 shows how this occurs for the oscillatory calcium signature (Fig. 2a) using the three binding affinities between the 4Ca^2+^–CaM–CAMTA complex and DNA, which are used in Fig. 6.

The oscillatory calcium signature (Fig. 2a) is nonlinearly amplified into a functional signal (4Ca^2+^–CaM–CAMTA complex) (Fig. 2c). In order to understand why the calcium signature induces gene expression in the specific ways described for Fig. 6, we calculate the potential steady‐state gene expression fold change by varying the 4Ca^2+^–CaM–CAMTA complex concentration (solid curve, Fig. 7) for three binding affinities between the 4Ca^2+^–CaM–CAMTA complex and DNA. The steady‐state gene expression fold change represents the maximum possible fold change in gene expression if time is sufficiently long so that steady‐state gene expression can become established for each 4Ca^2+^–CaM–CAMTA complex concentration. However, when a calcium signature emerges, the 4Ca^2+^–CaM–CAMTA complex concentration is a transient signal corresponding to the calcium signature and it does not establish a steady state (Figs 2c, 3c, 4c). Thus, actual gene expression follows the potential, but does not reach the potential, as explained in Fig. 7. At point I in Fig. 7(a), the oscillatory calcium signature has not emerged yet, and gene expression is at a steady state. When Ca^2+^ concentration elevates (Fig. 2a), the 4Ca^2+^–CaM–CAMTA complex concentration increases. At point II, gene expression has the potential to increase *c*. 800‐fold. However, the 4Ca^2+^–CaM–CAMTA complex concentration does not stay at point II and it starts to decrease from point II as a result of the decrease in Ca^2+^ concentration. At point III, gene expression has the potential to increase only *c*. 10‐fold. From point III to point IV, the 4Ca^2+^–CaM–CAMTA complex concentration continues to decrease following the calcium signature (Fig. 2a–c), and the potential fold increase of gene expression diminishes. At point IV, gene expression has no potential to increase at all, as the 4Ca^2+^–CaM–CAMTA complex concentration at point IV is the same as the original steady‐state concentration. From point I to point IV, gene expression continuously accumulates all the information from the calcium signature. During the first cycle of the calcium signature, gene expression increases to 1.6‐fold (Fig. 7a), although at point II it has the potential to increase *c*. 800‐fold and at point IV it has the potential to recover to the original steady‐state gene expression level, which is the level for which a calcium signature has not emerged. At point IV, gene expression memorizes the 1.6‐fold gene expression level and uses it as a starting point to read out the second cycle of the calcium signature (Fig. 2a). Gene expression response to the second cycle of the calcium signature follows the same principle as that for the first cycle. However, as gene expression memorizes the 1.6‐fold increase at point IV, at the end of the second cycle, it establishes a *c*. 1.9‐fold gene expression level (Fig. 7a). Again, the gene expression response memorizes this 1.9‐fold increase and continues to read out the third cycle of the calcium signature. After 10 cycles, gene expression increases to *c*. 4‐fold (point VI). At point VI, the calcium signature (Fig. 2a) ends and Ca^2+^ concentration recovers its original steady‐state level. Correspondingly, the 4Ca^2+^–CaM–CAMTA complex concentration also recovers its steady‐state level. Thus, at point VI, gene expression also starts to approach its original steady‐state level through point VII to point I. From point I to point VII, gene expression has continuously accumulated all the information during the lifetime of this calcium signature. Therefore, Fig. 7(a) reveals that the gene expression response depends on the history of the oscillatory calcium signature (Fig. 2a) and accumulates all information during the lifetime of this calcium signature.

Binding affinity between the 4Ca^2+^–CaM–CAMTA complex and DNA affects the dependence of the gene expression response on the history of a calcium signature. Fig. 7(b,c) show that, when the binding affinity is reduced or increased, the curve for the potential fold change of gene expression moves to the left or right, respectively. Thus, for the same oscillatory calcium signature (Fig. 2a), the reduced or increased binding affinity leads to larger (Fig. 7b) or smaller (Fig. 7c) gene expression fold changes, respectively. For example, when binding affinity is reduced (Fig. 7b), point II corresponds to a *c*. 8000‐fold potential gene expression change. However, when binding affinity is increased (Fig. 7c), point II corresponds to a *c*. 2‐fold potential gene expression change. This leads to a *c*. 16‐fold (Fig. 7b) and 1.006‐fold (Fig. 7c) actual gene expression change after the first cycle of the calcium signature, respectively. After 10 cycles of the calcium signature, a *c*. 115‐fold (Fig. 7b) or 1.03‐fold (Fig. 7c) actual gene expression change has been reached, respectively.

For both the transient calcium signature (Fig. 3a) and the prolonged calcium signature (Fig. 4a), how gene expression depends on the history of a calcium signature can also be analysed using the method summarized in Fig. 7. Specifically, gene expression accumulates information from both these calcium signatures in a similar manner to the first cycle of Fig. 7 (points I–IV), as shown in Figs S16 and S17. Therefore, for these two calcium signatures, gene expression also accumulates all information during their lifetimes. In summary, for all three types of calcium signatures (i.e. oscillatory (Fig. 2a), transient (Fig. 3a) and prolonged (Fig. 4a) calcium signatures), the gene expression response always depends on the history of the individual calcium signature and accumulates all information from the individual calcium signature, as shown in Figs 7, S16, S17. This explains phenomena such as why two signatures with equal areas under the curve (e.g. prolonged and transient) can give different gene expression responses (Whalley *et al*., 2011).

**Figure 8:**
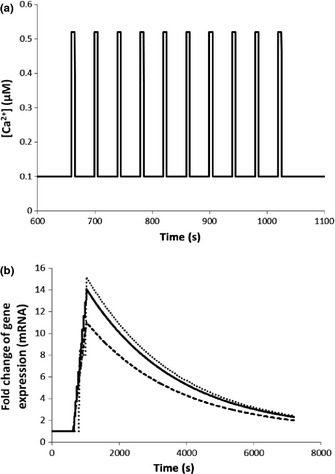
Fold change in gene expression induced by three piecewise calcium signatures that are reconstructed using the oscillatory calcium signature (Fig. 2a). Binding affinity (*K*_d_) between the active complex 4Ca^2+^–CaM–CAMTA and DNAis 1.1 × 10^−2^ μM. (a) The reconstructed piecewise calcium signature with *A* = 0.16 μM, [Ca^2+^]_max_ = 0.52 μM and [Ca^2+^]_min_ = 0.10 μM, *T* = 40 s. (b) Fold change in gene expression induced by three piecewise calcium signatures: bottom: *T* = 8 s (Fig. S18); middle: *T* = 40 s (a); top: *T* = 200 s (Fig. S19). CAMTA, calmodulin‐binding transcription activator; CaM, calmodulin.

The three calcium signatures (i.e. oscillatory (Fig. 2a), transient (Fig. 3a) and prolonged (Fig. 4a)) examined above have distinctive kinetics. We further investigate how the parameters in oscillations are linked to gene expression. To do so, we reconstruct piecewise calcium signatures using the oscillatory calcium signature (Fig. 2a). For a piecewise calcium signature, the following relationship is always valid.

(6) $$\begin{array}{cc} A= & \frac{t_{\max}{\left[{\text{Ca}}^{2+}\right]}_{\max}+t_{\min}{\left[{\text{Ca}}^{2+}\right]}_{\min}}{T}\\ T= & t_{\max}+t_{\min} \end{array}$$

(*A*, the average calcium concentration of the calcium signature; *t*_max_ and *t*_min_, the times for calcium concentration to be [Ca^2+^]_max_ and [Ca^2+^]_min_, respectively; *T*, the period.) For the oscillatory calcium signature (Fig. 2a) *A* = 0.16 μM and *T* = 40 s. The average maximum and minimum calcium concentrations of the 10 calcium spikes in Fig. 2(a) are [Ca^2+^]_max_ = 0.52 μM and [Ca^2+^]_min_ = 0.10 μM, respectively. Thus, using Eqn (6), a piecewise calcium signature is constructed (Fig. 8a). This piecewise calcium signature has the same key parameters (average, maximum and minimum calcium concentrations, periods and durations) as those in Fig. 2(a), but it has a piecewise shape (Fig. 8a). Similarly, we can use Eqn (6) to construct other piecewise calcium signatures with the same key parameters (average, maximum and minimum calcium concentrations and durations) as those in Fig. 2(a), but with different periods (Figs S18, S19). As a consequence of the difference in oscillatory period, for a duration of 400 s, a piecewise oscillatory calcium signature with a period of 8 s (Fig. S18), 40 s (Fig. 8a) and 200 s (Fig. S19) will contain 50, 10 and two spikes, respectively. Using the reconstructed three oscillatory calcium signatures, we have investigated how gene expression depends on both the shape and period of calcium signatures (Fig. 8b). First, Fig. 8(b) reveals that a piecewise calcium signature induces a larger fold change in gene expression than the oscillatory calcium signature in Fig. 2(a) (the curve for the gene expression induced by the reconstructed piecewise calcium signature with a period of 40 s in Fig. 8(b) is compared with the curve for the gene expression induced by an experimental calcium signature with a period of 40s in Fig. 6a). Second, gene expression fold change depends on the period of oscillatory piecewise calcium signatures. Specifically, at 1 h, a piecewise oscillatory calcium signature with a period of 8, 40 and 200 s induces a 4.8‐, 6.0‐ and 6.6‐fold change in gene expression, respectively. Thus, for a fixed duration, increasing the number of calcium spikes by decreasing the oscillatory period decreases the fold change of gene expression.

An alternative way to vary the number of calcium spikes is to alter the duration of a calcium signature while its oscillatory period is fixed. Fig. S20 shows that increasing the number of calcium spikes by increasing the duration of a calcium signature increases the fold change of gene expression. At 1 h, a piecewise calcium signature with 5, 40 and 75 spikes induces a 1.3‐, 4.0‐ and 6.9‐fold change in gene expression, respectively. Experimentally, it has been shown that nodulation gene expression is regulated by calcium spike number and the developmental status of the cell (Miwa *et al*., 2006). Combination of Figs 8(b) and S20 shows that calcium spike number is an important parameter regulating gene expression in our study. Moreover, both the oscillatory period and the duration of an oscillatory calcium signature also play their roles in gene expression (Figs 8b, S20). We note that our modelling results (Figs 8b, S20) are only applicable to CAMTA‐regulated gene expression and the gene expression mechanism we have used to calculate Figs 8(b) and S20 is the simple mechanism shown in Fig. 1. In general, gene expression may be regulated by other transcription factors and its expression mechanism may be different. In addition, Fig. 8(b) further indicates that the gene expression response represents an accumulation of all information on oscillatory periods in the three piecewise oscillatory calcium signatures during their lifetimes, as the only difference between the three calcium signatures (Figs 8a, S18, S19) is period.

## Discussion

Experimental data show that *A. thaliana* is able to decode different calcium signatures to produce specific gene expression responses (Whalley *et al*., 2011; Whalley & Knight, 2013). Some of these calcium‐dependent genes are targets for CAMTA. It is also known that CAMTAs have calmodulin‐binding domains (Finkler *et al*., 2007). Therefore, gene expression responses regulated by CAMTAs respond to calcium signals. In this work, we develop a modelling methodology that establishes the information flow from calcium signatures to CAMTA‐regulated gene expression. Specifically, the 4Ca^2+^–CaM–CAMTA interaction nonlinearly amplifies different calcium signatures. Then, amplification of Ca^2+^ signals allows the calcium signatures to be differentially decoded to give specific CAMTA‐regulated gene expression responses. Finally, mathematical modelling reveals that the gene expression response depends on the history of a calcium signature and accumulates all information during the lifetime of this calcium signature. This could account for why oscillations of different frequencies can activate different downstream calcium decoders, for example, CaM kinase II, rather than the amplitude and duration of spikes being responsible for the activation, as previously suggested (De Koninck & Schulman, 1998).

For plants to survive stress, it is vital that their responses are specific and appropriate to the particular stimulus. This means that the identity of the primary stimulus must be encoded in a ‘language’ the cell can understand. Most stimuli lead to transient elevations in cellular calcium concentrations. Importantly, different stimuli produce calcium elevations with different characteristics: a unique ‘calcium signature’. Consequently, the specific properties of different calcium signatures have been proposed to encode information on the identity of the stimulus (McAinsh *et al*., 1995; Allen *et al*., 2001; Love *et al*., 2004; Miwa *et al*., 2006; McAinsh & Pittman, 2009; Dodd *et al*., 2010; Short *et al*., 2012). For example, temperature stress responses are associated with specific calcium signatures (Knight & Knight, 2012). In plants, there are different mechanisms of Ca^2+^‐regulated gene expression (Kim *et al*., 2009; Galon *et al*., 2010; Bickerton & Pittman, 2012). One of the possible mechanisms is through the binding of Ca^2+^, CaM and transcription factors. Using the transcription factor CAMTA as an example, this work has developed a general methodology to establish the links between calcium signatures and gene expression. First, Ca^2+^ binds to its target proteins following the principles of thermodynamics. This process nonlinearly amplifies the Ca^2+^ signal. As the binding of Ca^2+^ to its target proteins may follow different binding mechanisms (Kim *et al*., 2009; Galon *et al*., 2010; Bickerton & Pittman, 2012), how different binding processes for Ca^2+^ and its target proteins amplify Ca^2+^ signals should be investigated for each type of transcription factor. As demonstrated in this work, a relatively small fold amplification of signal is able to induce a relatively large fold change in gene expression (Fig. 6b). If the binding affinity between the active complex 4Ca^2+^–CaM–CAMTA and DNA is further reduced from that in Fig. 6(b) (1.1 × 10^−3^ μM), any small fold amplification of signal is able to induce a relatively large fold change in gene expression. This demonstrates that any calcium signature, even a modest one, is able to induce gene expression. This explains how even very modest increases in cytosolic free calcium, for example, in response to ozone, can lead to increases in gene expression (Clayton *et al*., 1999). Moreover, different calcium signatures are thus capable of inducing specific gene expression patterns (Fig. 6). Second, which Ca^2+^ and protein binding complex is active for DNA binding should be experimentally explored. Based on experimental observation, the 4Ca^2+^–CaM complex is the active complex for Ca^2+^–CaM binding (Pifl *et al*., 1984). Moreover, the binding affinity between active complex and DNA should be measured, as modelling analysis reveals that it is a key parameter for specific gene expression responses to calcium signatures. Third, the mechanisms of gene expression should be investigated for all relevant genes. In particular, Ca^2+^ signals may affect several processes relating to gene expression. For example, it has been proposed that the expression of the genes downstream of Enhanced Disease Susceptibility 1 protein may be simultaneously positively and negatively regulated by calcium signals (Zhang *et al*., 2014). In addition, during symbiosis signalling, it has been shown that calcium/calmodulin‐dependent protein kinase is negatively and positively regulated by calcium (Miller *et al*., 2013). For these cases, although the gene expression mechanisms include multiple interaction points with Ca^2+^ signals, how Ca^2+^ signals affect gene expression can also be analysed using the methodology developed in this work. This can be done by introducing a more complex gene expression mechanism in the right pane of our Fig. 1. In the work presented here, as the gene expression mechanism is generally unknown, we use the simplest gene expression mechanisms (Fig. 1) to establish the links between calcium signatures and gene expression, demonstrating how different calcium signatures are decoded to produce specific CAMTA‐regulated gene expression responses. As actual gene expression mechanisms may be more complicated than those we used in this work, our results shown in Fig. 6 can only be qualitatively (not quantitatively) compared with our Table 2. The quantitative fold change of a specific gene should be further investigated if its expression mechanism and the related parameters are determined in the future. Finally, as the gene expression response accumulates all information during the lifetime of a calcium signature, it is important to accurately record the kinetics of a calcium signature during its lifetime. This work reveals that the information flow from calcium signatures to gene expression is an integrative dynamical system (Fig. 1) following thermodynamic principles. A combined experimental and modelling approach is able to establish this information flow. Based on the experimental data in Table 2, we assume that expression of all genes we have studied in this work is positively regulated by calcium signatures (no down‐regulated genes were empirically observed). Therefore, we use Eqn (4) to analyse gene expression. However, if expression of other (non‐CAMTA‐regulated) genes is negatively regulated by calcium signals, Eqn (5) should be used to analyse gene expression following the methodology established in this work. The same methodology can also be extended to analyse gene expression regulated by other Ca^2+^‐dependent transcription factors.

Parameterization of kinetic models is generally a challenging task (Liu *et al*., 2010; Almquist *et al*., 2014). In this work, we use the following process to parameterize the kinetic model (Fig. 1): (1) parameters that have been experimentally determined are used; (2) the relationship of parameters is constrained following thermodynamic principles; (3) the model sensitivity is evaluated by varying each of the adjustable parameters; (4) the model sensitivity is tested for certain parameter combinations by simultaneously varying all adjustable parameters. Our analysis shows that the modelling results presented in this work are robust to variations in the parameter values across a wide range. In addition, while our model has integrated a wide range of knowledge about Ca^2+^–CaM–CAMTA binding, many other aspects relating to Ca^2+^–CaM–CAMTA binding and activity have not been included in the current model. For instance, different CAMTA isoforms are expressed in different cell types (Mitsuda *et al*., 2003) and different CAMTA isoforms have been suggested to be involved in responses to different primary signals (Kim *et al*., 2013; Pandey *et al*., 2013; Benn *et al*., 2014). It is also not known whether CAMTAs are subject to posttranslational modifications, so this feature is also not included in our model. Therefore, we consider the current model to be a starting point for establishing the relationship between calcium signatures and gene expression responses.

Mathematical modelling is an important tool with which to establish the link between stimulus, calcium signatures and gene expression. Currently, modelling analysis concentrates on different aspects of this link. For example, this work establishes the link between calcium signatures and gene expression for CAMTA‐regulated genes in *A. thaliana* cells. For other cells such as hepatocytes, various modelling efforts have also been made in an attempt to understand the decoding of calcium signals (Larsen *et al*., 2004; Schuster *et al*., 2005; Dupont *et al*., 2011). Information transfer in Ca^2+^ signalling pathways was also studied by combining experimental data and mathematical modelling (Pahle *et al*., 2008). In plant cells, other modelling work includes how different calcium signatures are generated from different stimuli. Specifically, a simple model for the cytosolic pool was used to explain the generation of calcium signatures by assuming that calcium‐permeable channels depend solely on the cooling rate and that calcium pumps are dependent on the absolute temperature (Plieth, 1999). A model of action potential in cells of vascular plants for the cytosolic pool was developed by incorporating K^+^, Cl^−^ and Ca^2+^ channels; H^+^ and Ca^2+^ ATPases; the 2H^+^/Cl^−^ symporter; and the H^+^/K^+^ antiporter. The model supports a hypothesis that H^+^ ATPase participates in AP generation (Vladimir & Vladimir, 2009). Recently, an integrative model that incorporates the interactions of Ca^2+^, H^+^, K^+^, Cl^−^ and ATP in both cytosolic and vacuolar pools reveals how multiple ions in both the cytosol and the vacuole interplay to shape low‐temperature calcium signatures in plant cells (Liu *et al*., 2012). In addition, noisy time series of calcium oscillations (Granqvist *et al*., 2011) and generation of calcium signatures in other subcellular compartments such as the nucleus (Granqvist *et al*., 2012) have been studied. All of these studies and other modelling work in plant cells made efforts to establish links between stimuli and calcium signatures. Thus, it is plausible that, by integrating the links between stimuli and calcium signatures in the literature with the links between calcium signatures and gene expression response as described in this work, future research will be able to establish the relationship of stimuli, calcium signatures and gene expression responses. Thus, an integrative view of calcium signalling in plant cells can be formulated by integrating modelling and experimental studies.

## Supporting information

Please note: Wiley Blackwell are not responsible for the content or functionality of any supporting information supplied by the authors. Any queries (other than missing material) should be directed to the *New Phytologist* Central Office.

**Fig. S1** The effects of varying the *K*_d_ of R33, *K*_d(*R*33)_, by changing *k*_on_.
**Fig. S2** The effects of varying the *K*_d_ of R33, *K*_d(*R*33)_, by changing *k*_off_.
**Fig. S3** The effects of varying the *K*_d_ of R20, *K*_d(*R*20)_, by changing *k*_on_.
**Fig. S4** The effects of varying the *K*_d_ of R20, *K*_d(*R*20)_, by changing *k*_off_.
**Fig. S5** The effects of varying the cooperative binding between CaM and CAMTA in the presence of Ca^2+^.
**Fig. S6** The effects of varying the total CaM concentration on the amplification of Ca^2+^ signals.
**Fig. S7** The effects of simultaneously varying all five adjustable parameters (example 1).
**Fig. S8** The effects of simultaneously varying all five adjustable parameters (example 2).
**Fig. S9** Dependence of fold change in gene expression induced by an oscillatory calcium signature (Fig. 2a) on the delay time.
**Fig. S10** Dependence of fold change in gene expression induced by a transient calcium signature (Fig. 3a) on the delay time.
**Fig. S11** Dependence of fold change in gene expression induced by a prolonged calcium signature (Fig. 4a) on the delay time.
**Fig. S12** The effects of varying the base rate for gene transcription (*k*_1_) on the fold change of gene expression.
**Fig. S13** The effects of varying the maximal rate for 4Ca^2+^–CaM–CAMTA complex‐regulated gene transcription (*k*_2_) on fold change of gene expression.
**Fig. S14** The effects of varying the Hill coefficient (*n*) on fold change of gene expression.
**Fig. S15** The effects of varying the decay constant of mRNA (*k*_3_) on fold change of gene expression.
**Fig. S16** Gene expression accumulates all information during the lifetime of the transient calcium signature (Fig. 3a).
**Fig. S17** Gene expression accumulates all information during the lifetime of the prolonged calcium signature (Fig. 4a).
**Fig. S18** The reconstructed piecewise calcium signature with *T* = 8 s.
**Fig. S19** The reconstructed piecewise calcium signature with *T* = 200 s.
**Fig. S20** Effects of the number of calcium spikes on fold change in gene expression.
**Notes S1** Modelling equations.
