# Supplementary material for: Combining modelling and experimental approaches to explain how calcium signatures are decoded by calmodulin‐binding transcription activators (CAMTAs) to produce specific gene expression responses
Source: New Phytol. 2015 Apr 27;208(1):174–87. doi: 10.1111/nph.13428 (PMC4832281; doi:10.1111/nph.13428)
Supplement: Supplementary file 1 — Fig. S1 The effects of varying the K d of R33, K d(R33), by changing k on. Fig. S2 The effects of varying the K d of R33, K d(R33), by changing k off. Fig. S3 The effects of varying the K d of R20, K d(R20), by changing k on. Fig. S4 The effects of varying the K d of R20, K d(R20), by changing k off. Fig. S5 The effects of varying the cooperative binding between CaM and CAMTA in the presence of Ca2+. Fig. S6 The effects of varying the total CaM concentration on the amplification of Ca2+ signals. Fig. S7 The effects of simultaneously varying all five adjustable parameters (example 1). Fig. S8 The effects of simultaneously varying all five adjustable parameters (example 2). Fig. S9 Dependence of fold change in gene expression induced by an oscillatory calcium signature (Fig. 2a) on the delay time. Fig. S10 Dependence of fold change in gene expression induced by a transient calcium signature (Fig. 3a) on the delay time. Fig. S11 Dependence of fold change in gene expression induced by a prolonged calcium signature (Fig. 4a) on the delay time. Fig. S12 The effects of varying the base rate for gene transcription (k 1) on the fold change of gene expression. Fig. S13 The effects of varying the maximal rate for 4Ca2+–CaM–CAMTA complex‐regulated gene transcription (k 2) on fold change of gene expression. Fig. S14 The effects of varying the Hill coefficient (n) on fold change of gene expression. Fig. S15 The effects of varying the decay constant of mRNA (k 3) on fold change of gene expression. Fig. S16 Gene expression accumulates all information during the lifetime of the transient calcium signature (Fig. 3a). Fig. S17 Gene expression accumulates all information during the lifetime of the prolonged calcium signature (Fig. 4a). Fig. S18 The reconstructed piecewise calcium signature with T = 8 s. Fig. S19 The reconstructed piecewise calcium signature with T = 200 s. Fig. S20 Effects of the number of calcium spikes on fold change in gene expression. Notes S1 Modelling equations. [file NPH-208-174-s001.pdf]

New Phytologist **Supporting Information Figs S1-S20 and Notes S1**

Combining modelling and experimental approaches to explain how calcium signatures are decoded by CAMTA to produce specific gene expression responses

Authors: Junli Liu, Helen J. Whalley & Marc R. Knight

Article acceptance date: 26 March 2015

The following Supporting Information is available for this article:

Fig. S1 The effects of varying K_d of R33, $K_{d(R33)}$, by changing kon.

Fig. S2 The effects of varying K_d of R33, $K_{d(R33)}$, by changing koff.

Fig. S3 The effects of varying K_d of R20, $K_{d(R20)}$, by changing kon.

Fig. S4 The effects of varying K_d of R20, $K_{d(R20)}$, by changing koff.

Fig. S5 The effects of varying the cooperative binding between CaM and CAMTA in the presence of Ca^{2+}.

Fig. S6 The effects of varying the total CaM concentration on the amplification of Ca^{2+} signals.

Fig. S7 The effects of simultaneously varying all five adjustable parameters (example 1).

Fig. S8 The effects of simultaneously varying all five adjustable parameters (example 2).

Fig. S9 Dependence of fold change in gene expression induced by oscillatory calcium signature (Fig. 2a) on the delay time.

Fig. S10 Dependence of fold change in gene expression induced by transient calcium signature (Fig. 3a) on the delay time.

Fig. S11 Dependence of fold change in gene expression induced by prolonged calcium signature (Fig. 4a) on the delay time.

Fig. S12 The effects of varying base rate for gene transcription (k_1) on fold change of gene expression.

Fig. S13 The effects of varying maximal rate for $4Ca^{2+}$-CaM-CAMTA complex-regulated gene transcription (k_2) on fold change of gene expression.

Fig. S14 The effects of varying Hill coefficient (n) on fold change of gene expression.

Fig. S15 The effects of varying the decay constant of mRNA (k_3) on fold change of gene expression.

Fig. S16 Gene expression accumulates all information during the lifetime of the transient calcium signature (Fig. 3a).

Fig. S17 Gene expression accumulates all information during the lifetime of the prolonged calcium signature (Fig. 4a).

Fig. S18 The reconstructed piecewise calcium signature with T=8s.

Fig. S19 The reconstructed piecewise calcium signature with T=200s.

Fig. S20 Effects of the number of calcium spikes on fold change in gene expression

Notes S1 Modelling equations.

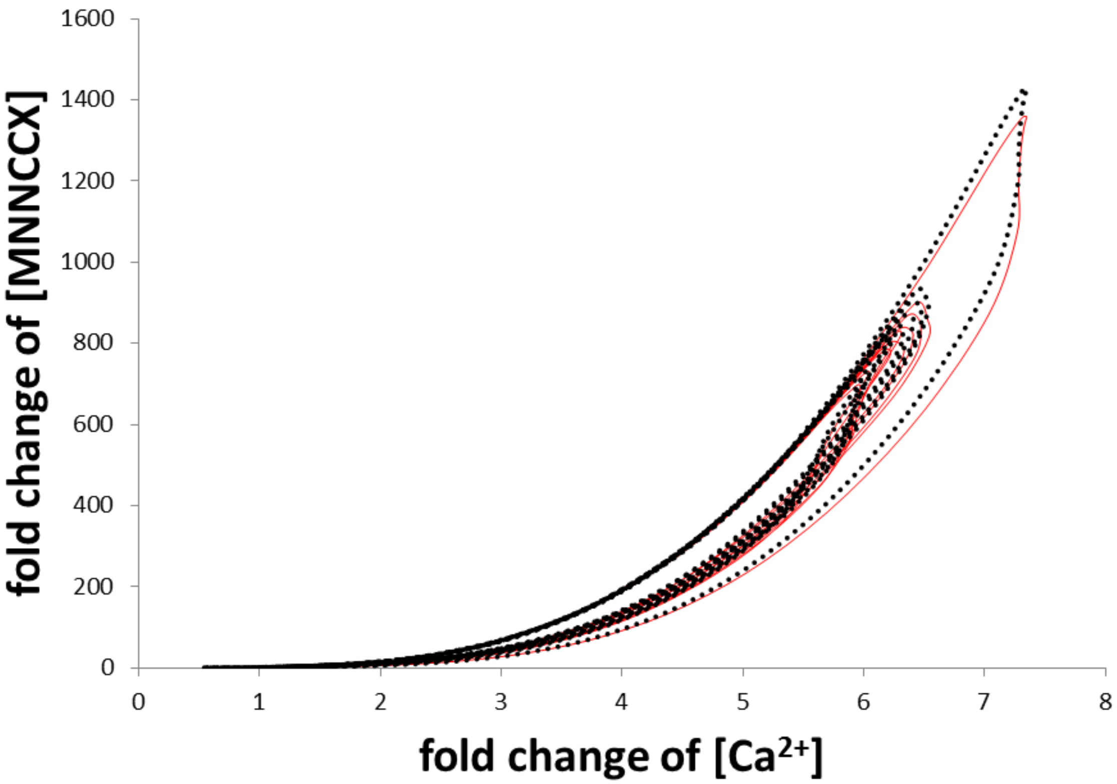

Fig. S1 The effects of varying K_d of R33, $K_{d(R33)}$, by changing kon. When $K_{d(R33)}$ changes, the following relationship must be valid due to thermodynamic constraints: $K_{d(R12)}K_{d(R33)} = K_{d(R16)}K_{d(R32)}$; $K_{d(R8)}K_{d(R33)} = K_{d(R19)}K_{d(R28)}$ and $K_{d(R30)}K_{d(R28)} = K_{d(R26)}K_{d(R32)}$. In order to maintain the above relationships, $K_{d(R32)}$ and $K_{d(R28)}$ are increased or decreased by same fold as that of $K_{d(R33)}$. The change of $K_{d(R32)}$ and $K_{d(R28)}$ is realised by changing their respective kon. Solid red lines: $K_{d(R33)}$ is increased by 10 folds by changing kon, as binding of Ca^{2+}-CaM complex to CAMTA is tighter than binding of free CaM to CAMTA. Dashed black line: $K_{d(R33)}$ is decreased by 100 folds by changing kon. The reference value is $K_{d(R33)}$ =1.2e-3 μM (Fig. 2).

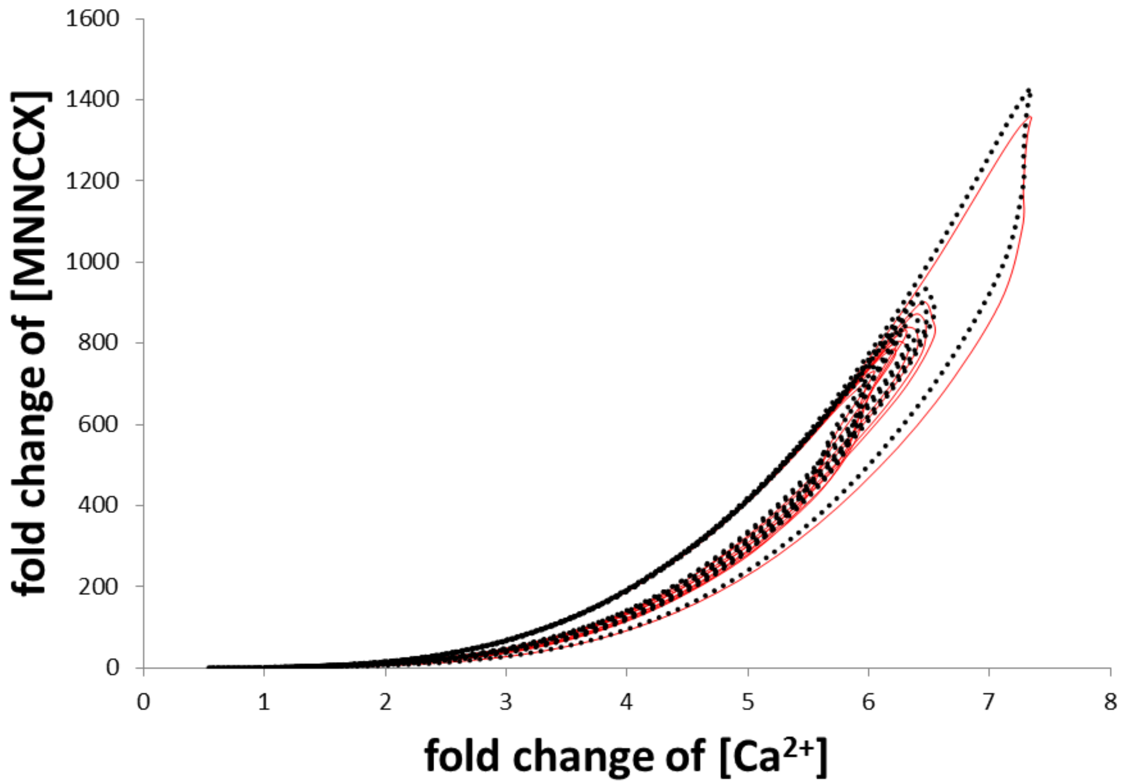

Fig. S2 The effects of varying K_d of R33, $K_{d(R33)}$, by changing koff. When $K_{d(R33)}$ changes, the following relationship must be valid due to thermodynamic constraints: $K_{d(R12)}K_{d(R33)} = K_{d(R16)}K_{d(R32)}$; $K_{d(R8)}K_{d(R33)} = K_{d(R19)}K_{d(R28)}$ and $K_{d(R30)}K_{d(R28)} = K_{d(R26)}K_{d(R32)}$. In order to maintain the above relationships, $K_{d(R32)}$ and $K_{d(R28)}$ are increased or decreased by same fold as that of $K_{d(R33)}$. The change of $K_{d(R32)}$ and $K_{d(R28)}$ is realised by changing their respective kon. Solid red lines: $K_{d(R33)}$ is increased by 10 folds by changing koff, as binding of Ca^{2+}–CaM complex to CAMTA is tighter than binding of free CaM to CAMTA. Dashed black line: $K_{d(R33)}$ is decreased by 100 folds by changing koff. The reference value is $K_{d(R33)}$=1.2e-3 μM (Fig. 2).

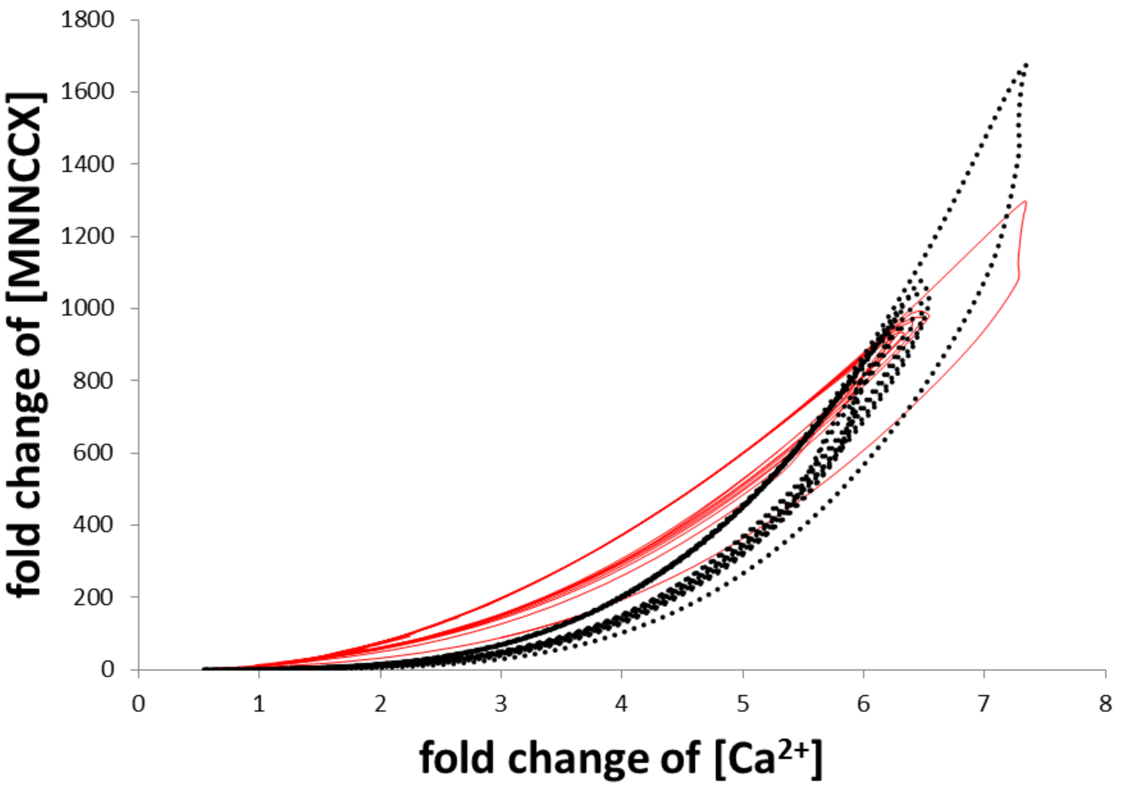

Fig. S3 The effects of varying K_d of R20, $K_{d(R20)}$, by changing kon. When $K_{d(R20)}$ changes, the following relationship must be valid due to thermodynamic constraints: $K_{d(R20)}K_{d(R27)} = K_{d(R7)}K_{d(R19)}$; $K_{d(R22)}K_{d(R27)} = K_{d(R25)}K_{d(R30)}$ and $K_{d(R2)}K_{d(R20)} = K_{d(R18)}K_{d(R22)}$. In order to maintain the above relationships, when $K_{d(R20)}$ is increased, $K_{d(R22)}$ and $K_{d(R27)}$ are increased and decreased by same fold as that of $K_{d(R20)}$ respectively. When $K_{d(R20)}$ is decreased, $K_{d(R22)}$ and $K_{d(R27)}$ are decreased and increased by same fold as that of $K_{d(R20)}$ respectively. The change of $K_{d(R22)}$ and $K_{d(R27)}$ is realised by changing their respective kon. Solid red lines: $K_{d(R20)}$ is increased by 10 folds by changing kon, as binding of Ca^{2+}–CaM complex to CAMTA is tighter than binding of free CaM to CAMTA. Dashed black line: $K_{d(R20)}$ is decreased by 100 folds by changing kon. The reference value is $K_{d(R20)}$ =1.2e-3 μM (Fig. 2).

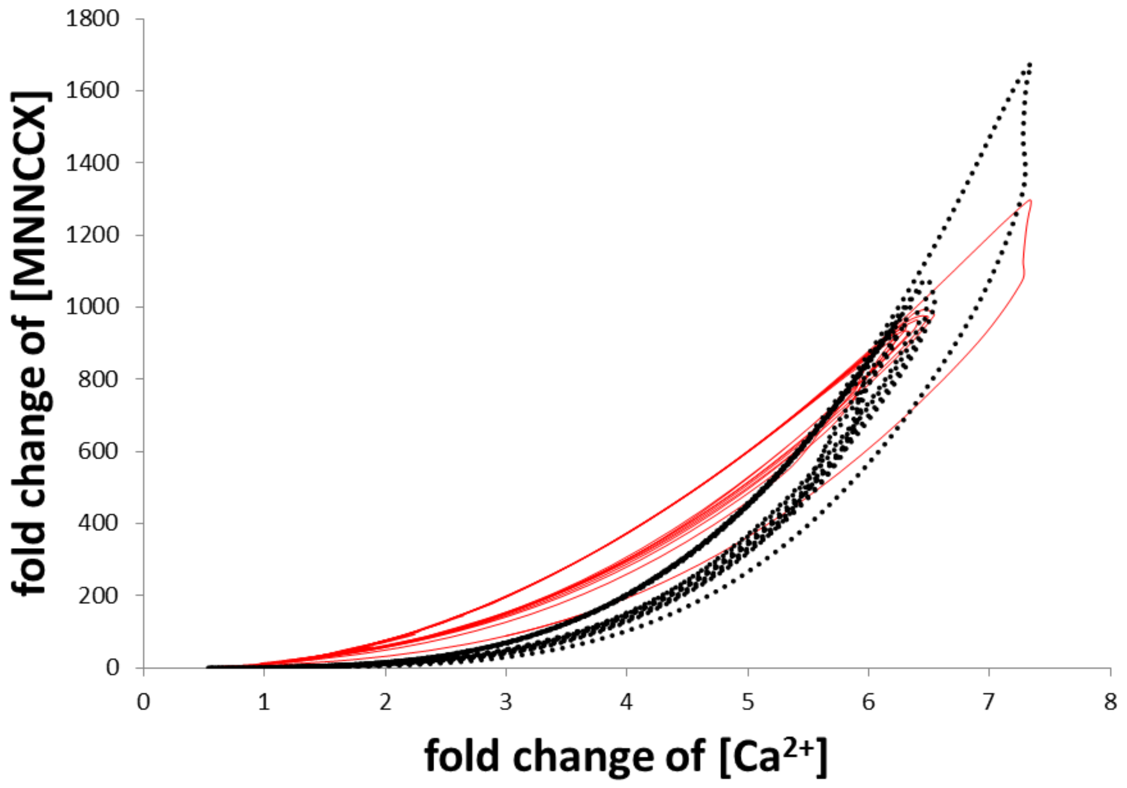

Fig. S4 The effects of varying K_d of R20, $K_{d(R20)}$, by changing koff. When $K_{d(R20)}$ changes, the following relationship must be valid due to thermodynamic constraints: $K_{d(R20)}K_{d(R27)} = K_{d(R7)}K_{d(R19)}$; $K_{d(R22)}K_{d(R27)} = K_{d(R25)}K_{d(R30)}$ and $K_{d(R2)}K_{d(R20)} = K_{d(R18)}K_{d(R22)}$. In order to maintain the above relationships, when $K_{d(R20)}$ is increased, $K_{d(R22)}$ and $K_{d(R27)}$ are increased and decreased by same fold as that of $K_{d(R20)}$ respectively. When $K_{d(R20)}$ is decreased, $K_{d(R22)}$ and $K_{d(R27)}$ are decreased and increased by same fold as that of $K_{d(R20)}$ respectively. The change of $K_{d(R22)}$ and $K_{d(R27)}$ is realised by changing their respective koff. Solid red lines: $K_{d(R20)}$ is increased by 10 folds by changing kon, as binding of Ca^{2+}–CaM complex to CAMTA is tighter than binding of free CaM to CAMTA. Dashed black line: $K_{d(R20)}$ is decreased by 100 folds by changing koff. The reference value is $K_{d(R20)}$ =1.2e-3 μM (Fig. 2).

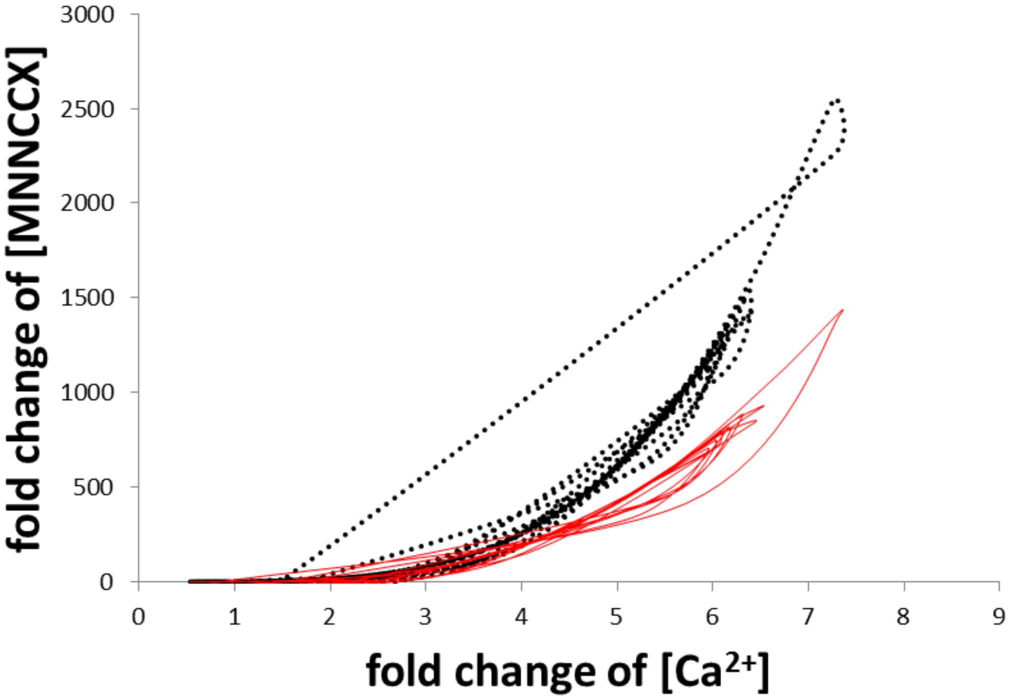

Fig. S5 The effects of varying the cooperative binding between CaM and CAMTA in the presence of Ca^{2+} (P in Eqn 2 in the main text). Solid red lines: P=1.0. Dashed black line: P=1.0E-3. The reference value is P=0.1 (Fig. 2).

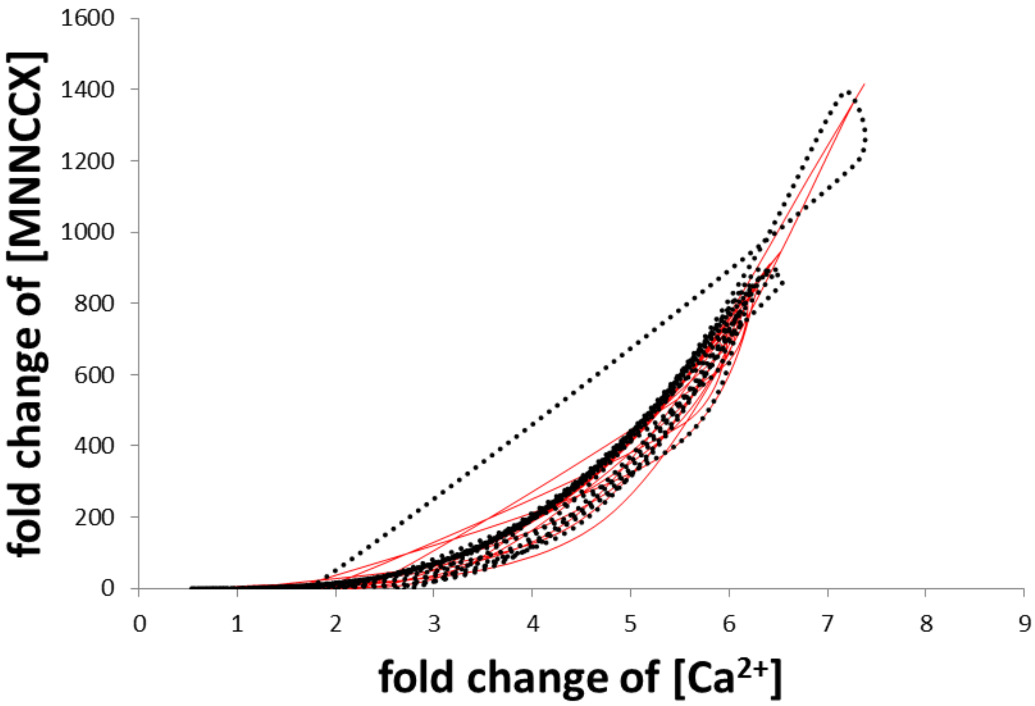

Fig. S6 The effects of varying the total CaM concentration on the amplification of Ca^{2+} signals. Solid red line: total CaM concentration is 1000 μM. Dashed black line: total CaM concentration is 0.1 μM. The reference value is 10 μM (Fig. 2).

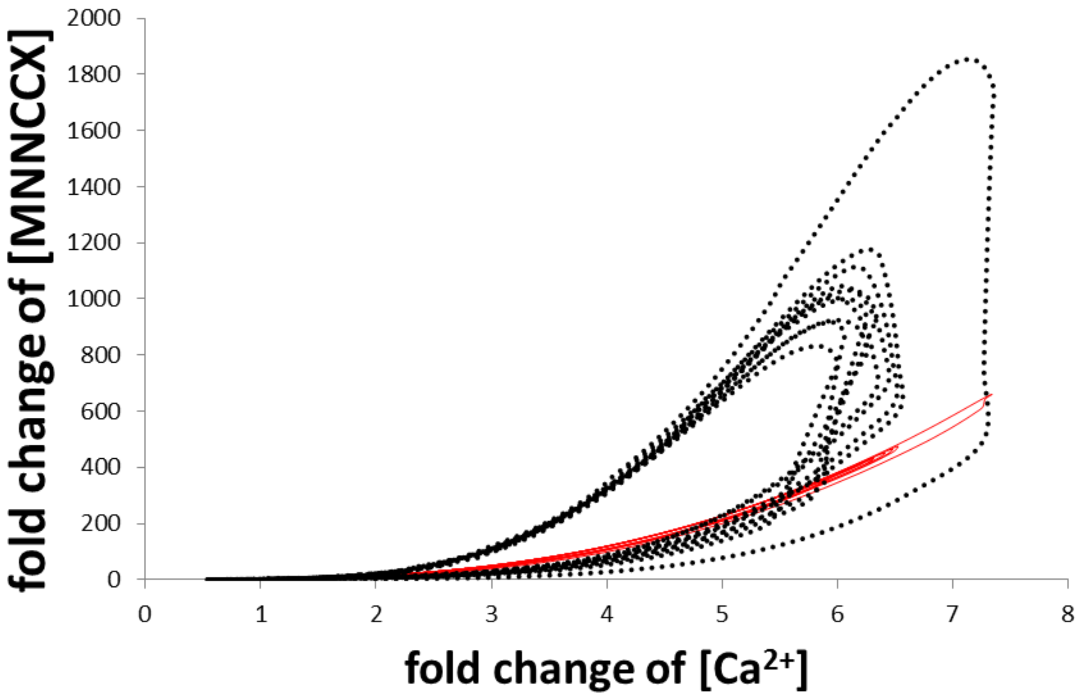

Fig. S7 The effects of simultaneously varying all five adjustable parameters (example 1). Solid red line: total CaM concentration is 1000 μM, total CAMTA concentration: 1000 μM , the cooperative binding between CaM and CAMTA in the presence of Ca^{2+}due to on binding rate (Q in Eqn 3): 100, the cooperative binding between CaM and CAMTA in the presence of Ca^{2+} (P in Eqn 2): 0.01, on rate for the binding between the Ca^{2+}–CaM complex and CAMTA ($k_{on(R14)}$): 100 $\mu M^{-1} s^{-1}$. Dashed black line: total CaM concentration is 0.1 μM, total CAMTA concentration: 0.1 μM , the cooperative binding between CaM and CAMTA in the presence of Ca^{2+}due to on binding rate (Q in Eqn 3): 0.01, the cooperative binding between CaM and CAMTA in the presence of Ca^{2+} (P in Eqn 2): 1, on rate for the binding between the Ca^{2+}–CaM complex and CAMTA ($k_{on(R14)}$): 0.01 $\mu M^{-1} s^{-1}$. Reference value (Fig. 2): total CaM concentration is 10 μM, total CAMTA concentration: 10 μM , the cooperative binding between CaM and CAMTA in the presence of Ca^{2+}due to on binding rate (Q in Eqn 3): 1, the cooperative binding between CaM and CAMTA in the presence of Ca^{2+} (P in Eqn 2): 0.1, on rate for the binding between the Ca^{2+}–CaM complex and CAMTA ($k_{on(R14)}$): 1 $\mu M^{-1} s^{-1}$.

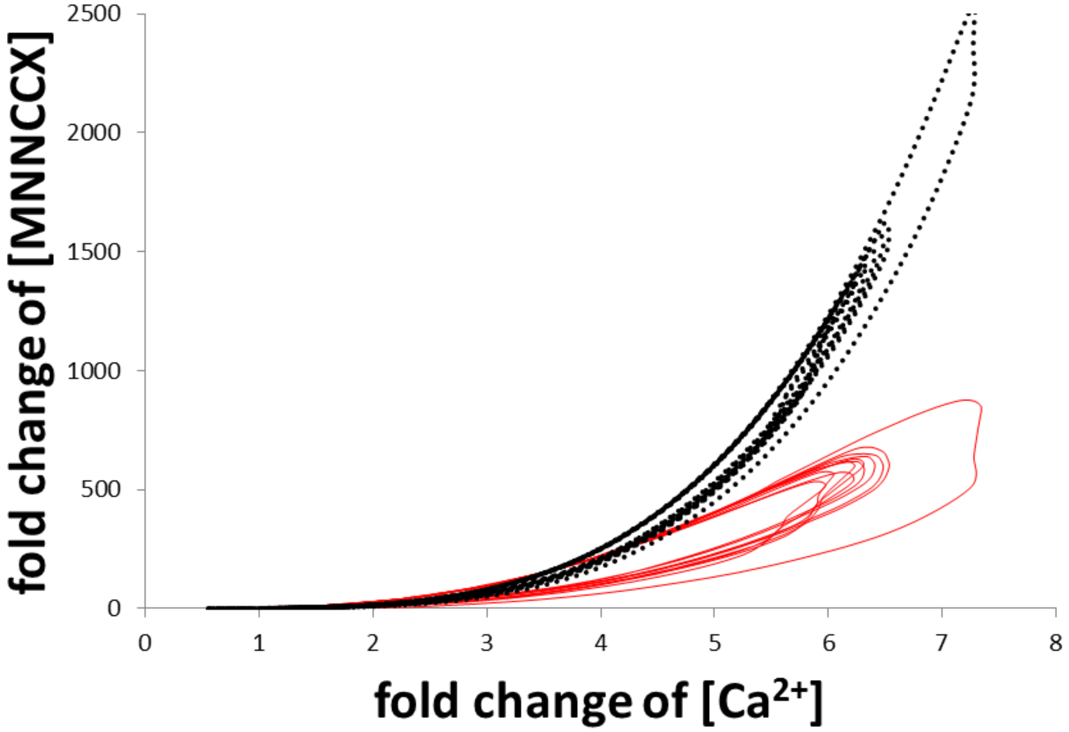

Fig. S8 The effects of simultaneously varying all five adjustable parameters (example 2). Solid red line: total CaM concentration is 0.1 µM, total CAMTA concentration: 1000 µM, the cooperative binding between CaM and CAMTA in the presence of Ca^{2+}due to on binding rate (Q in Eqn 3): 0.01, the cooperative binding between CaM and CAMTA in the presence of Ca^{2+} (P in Eqn 2): 0.01, on rate for the binding between the Ca^{2+}–CaM complex and CAMTA ($k_{on(R14)}$): 100 $\mu M^{-1}s^{-1}$. Dashed black line: total CaM concentration is 1000 µM, total CAMTA concentration: 0.1 µM , the cooperative binding between CaM and CAMTA in the presence of Ca^{2+}due to on binding rate (Q in Eqn 3): 100, the cooperative binding between CaM and CAMTA in the presence of Ca^{2+} (P in Eqn 2): 1, on rate for the binding between the Ca^{2+}–CaM complex and CAMTA ($k_{on(R14)}$): 0.01 $\mu M^{-1}s^{-1}$. Reference value (Fig. 2): total CaM concentration is 10 µM, total CAMTA concentration: 10 µM , the cooperative binding between CaM and CAMTA in the presence of Ca^{2+}due to on binding rate (Q in Eqn 3): 1, the cooperative binding between CaM and CAMTA in the presence of Ca^{2+} (P in Eqn 2): 0.1, on rate for the binding between the Ca^{2+}–CaM complex and CAMTA ($k_{on(R14)}$): 1 $\mu M^{-1}s^{-1}$.

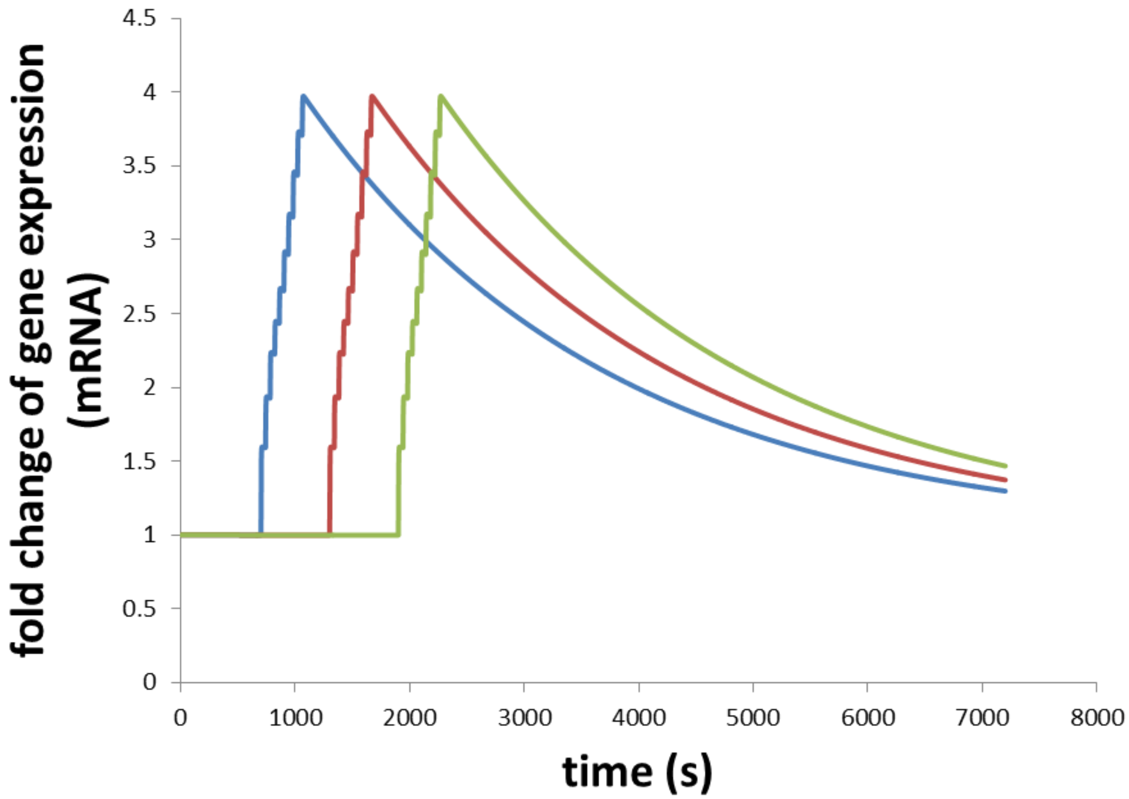

Fig. S9 Dependence of fold change in gene expression induced by oscillatory calcium signature (Fig. 2a) on the delay time. Binding affinity (K_d) is 1.1e-2 µM. Blue line: Delay time is 600 s (reference value (Fig. 6)). Red line: Delay time is 1200 s. Green line: Delay time is 1800 s.

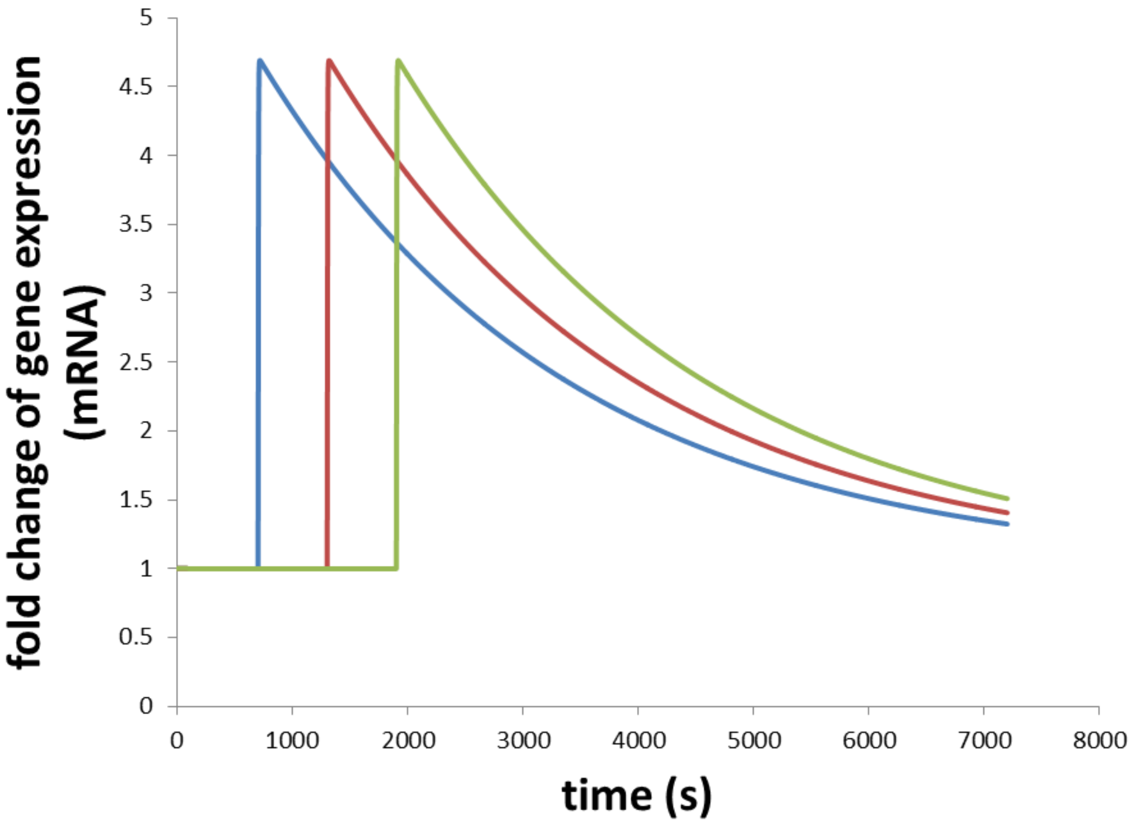

Fig. S10 Dependence of fold change in gene expression induced by transient calcium signature (Fig. 3a) on the delay time. Binding affinity (K_d) is 1.1e-2 µM. Blue line: Delay time is 600 s (reference value (Fig. 6)). Red line: Delay time is 1200 s. Green line: Delay time is 1800 s.

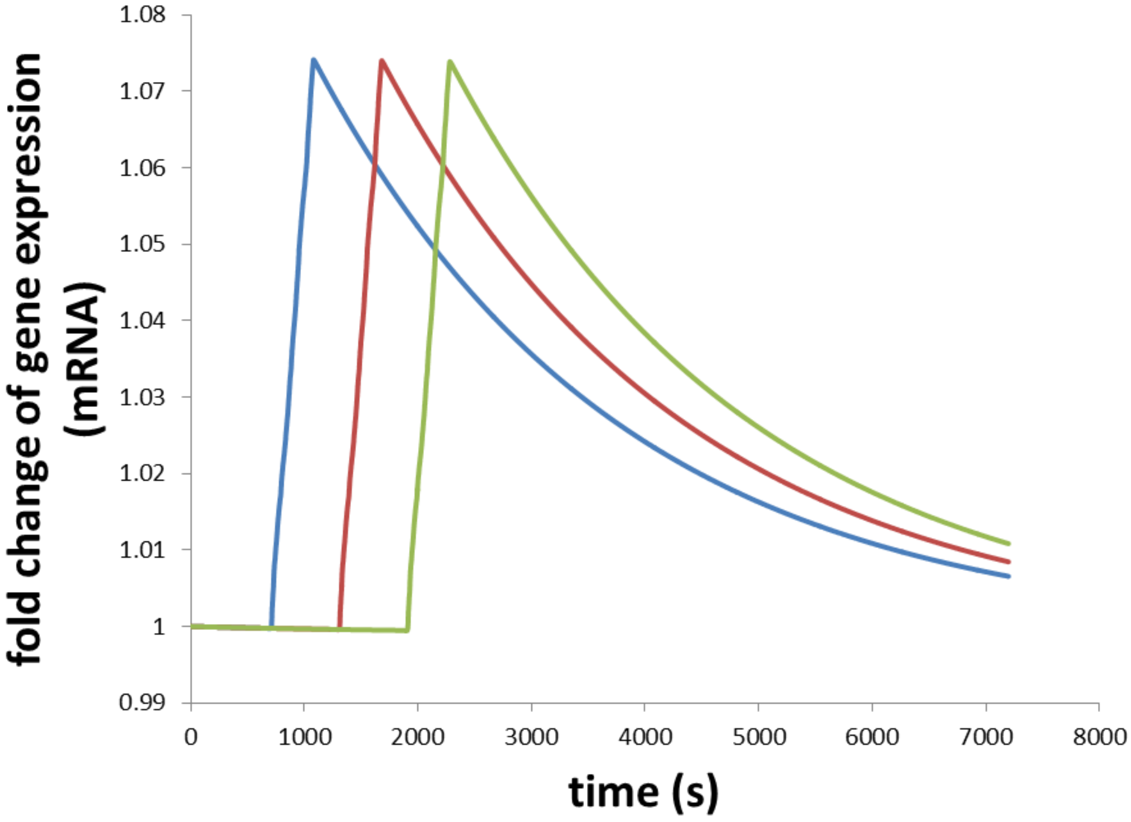

Fig. S11 Dependence of fold change in gene expression induced by prolonged calcium signature (Fig. 4a) on the delay time. Binding affinity (K_d) is 1.1e-2 µM. Blue line: Delay time is 600 s (reference value (Fig. 6)). Red line: Delay time is 1200 s. Green line: Delay time is 1800 s.

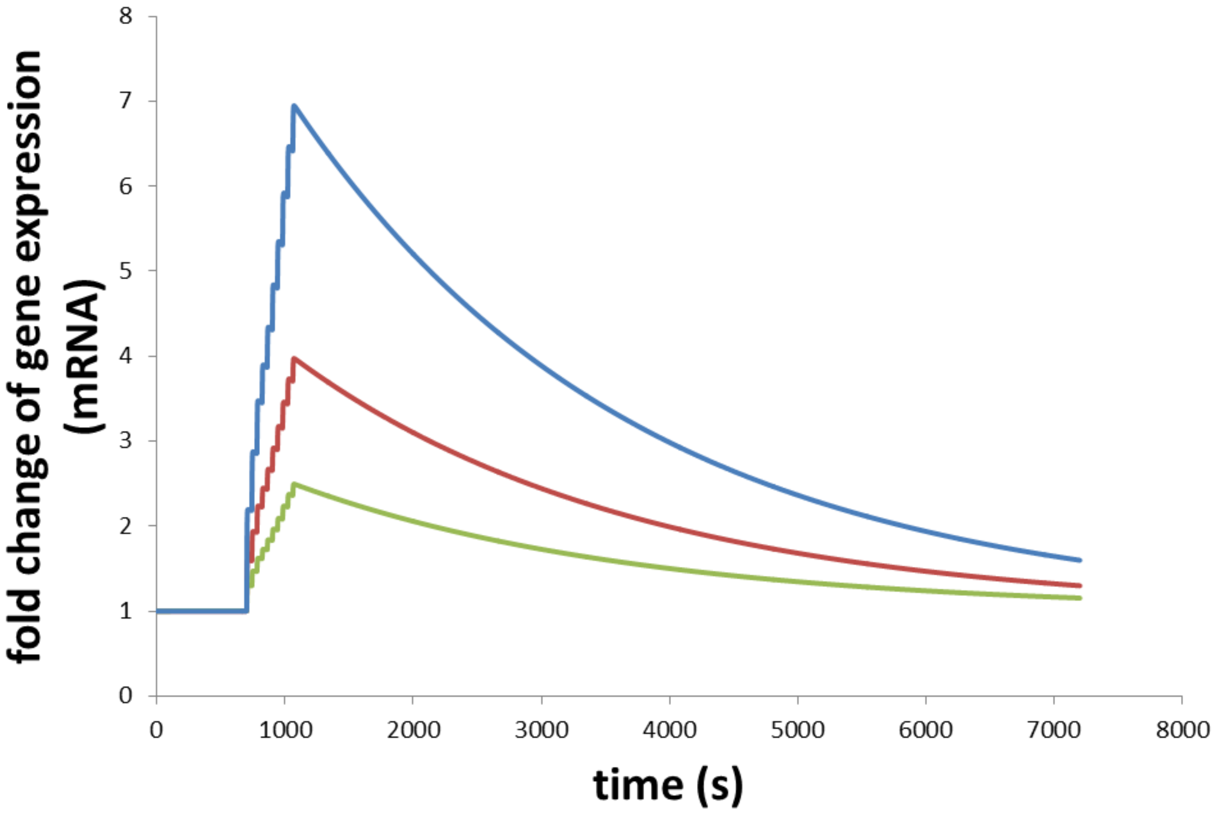

Fig. S12 The effects of varying base rate for gene transcription (k_1) on fold change of gene expression. Binding affinity (K_d) is 1.1e-2 μM. Red line: $k_1 = 5.0 \times 10^{-6}$ μM s^{-1} (reference value (Fig. 6)). Blue line: $k_1 = 1.0 \times 10^{-5}$ μM s^{-1}. Green line: $k_1 = 2.5 \times 10^{-6}$ μM s^{-1}.

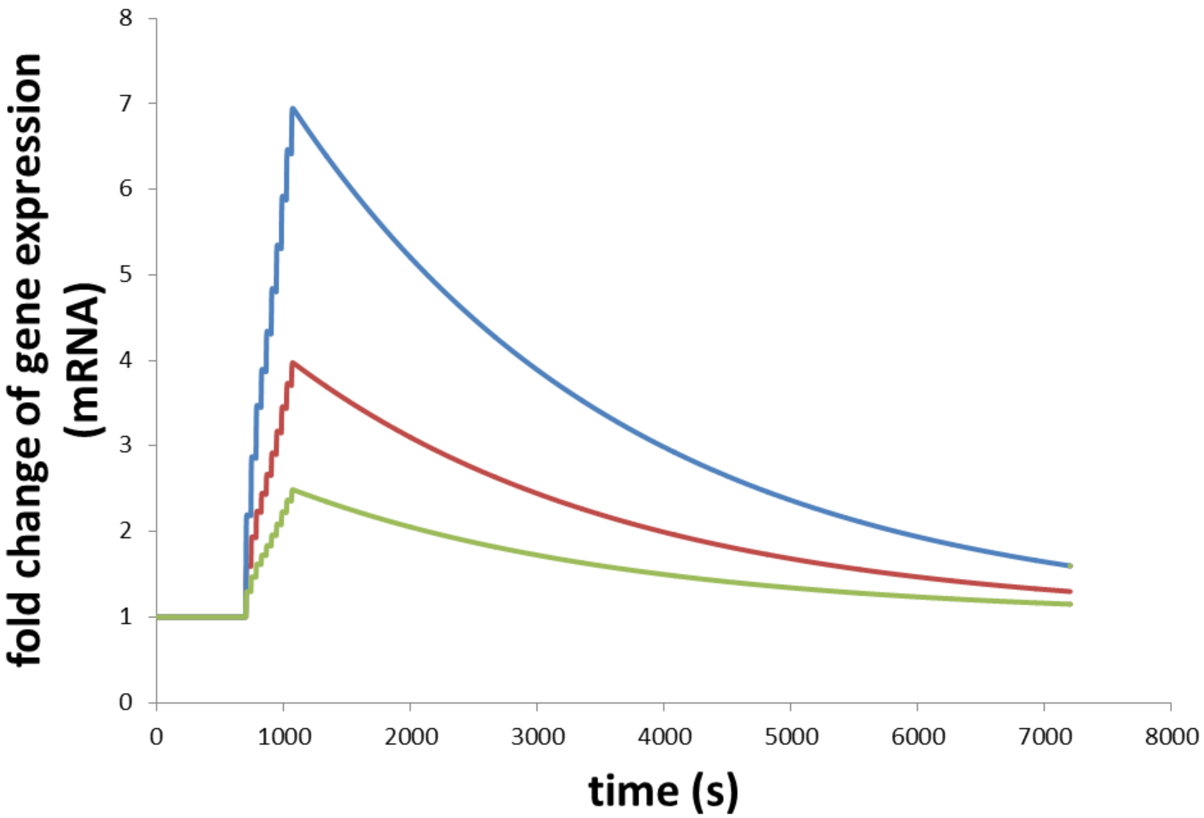

Fig. S13 The effects of varying maximal rate for $4Ca^{2+}$–CaM–CAMTA complex-regulated gene transcription (k_2) on fold change of gene expression. Binding affinity (K_d) is 1.1e-2 μM. Red line: $k_2 = 5.0 \times 10^{-2}$ μM s^{-1} (reference value (Fig. 6)). Blue line: $k_2 = 0.1$ μM s^{-1}. Green line: $k_2 = 2.5 \times 10^{-2}$ μM s^{-1}.

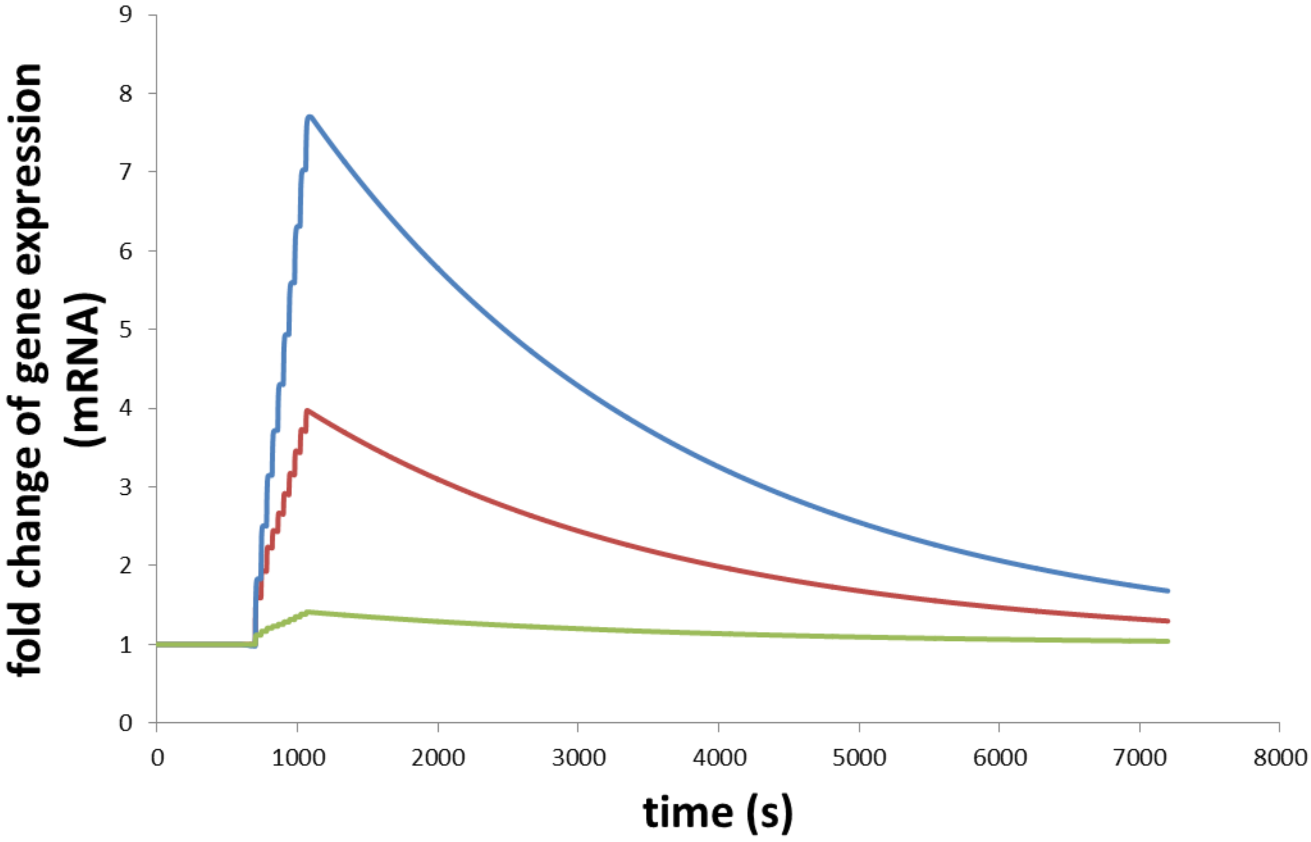

Fig. S14 The effects of varying Hill coefficient (n) on fold change of gene expression. Binding affinity (K_d) is 1.1e-2 μM. Red line: n=2 (reference value (Fig. 6)). Blue line: n=1. Green line: n=3.

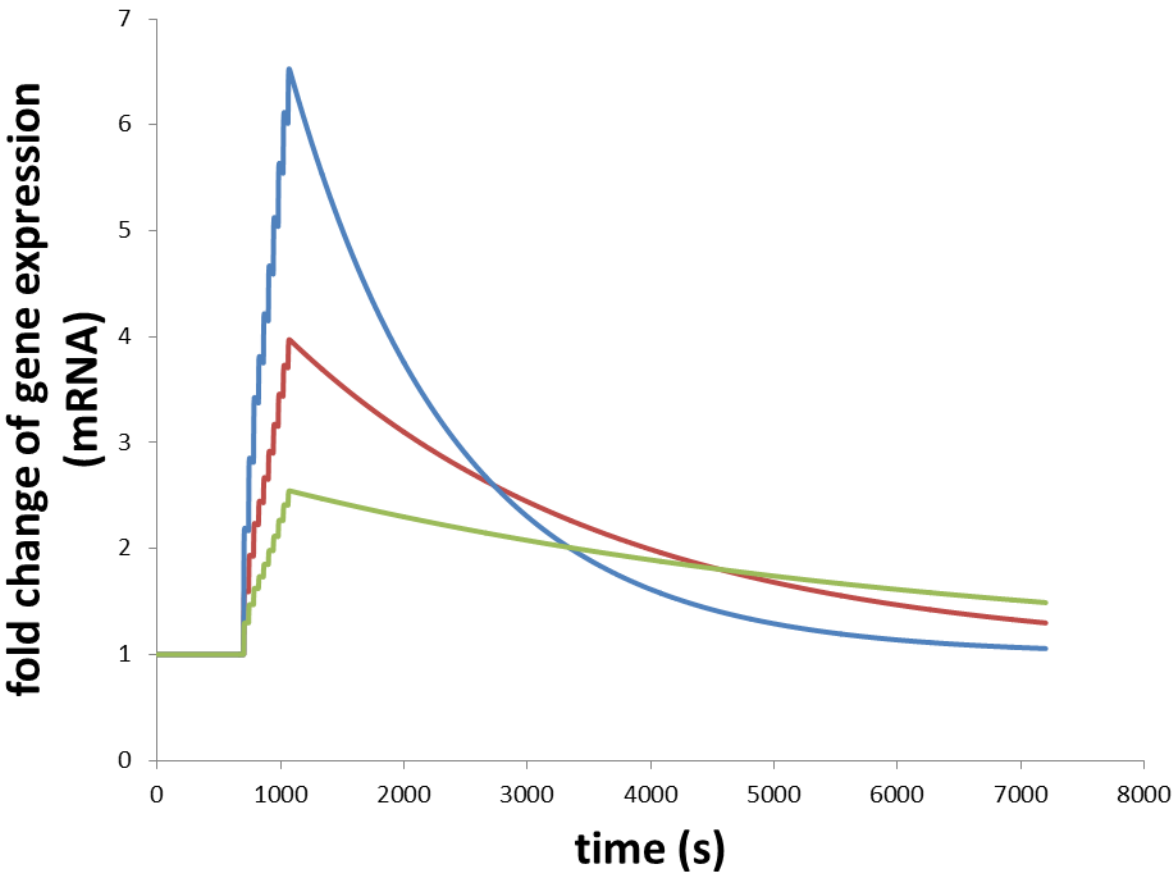

Fig. S15 The effects of varying the decay constant of mRNA (k_3) on fold change of gene expression. Binding affinity (K_d) is 1.1e-2 μM. Red line: $k_3 = 3.75 \times 10^{-4}$ s^{-1} (reference value (Fig. 6)). Blue line: $k_3 = 7.5 \times 10^{-4}$ s^{-1}. Green line: $k_3 = 1.875 \times 10^{-4}$ s^{-1}.

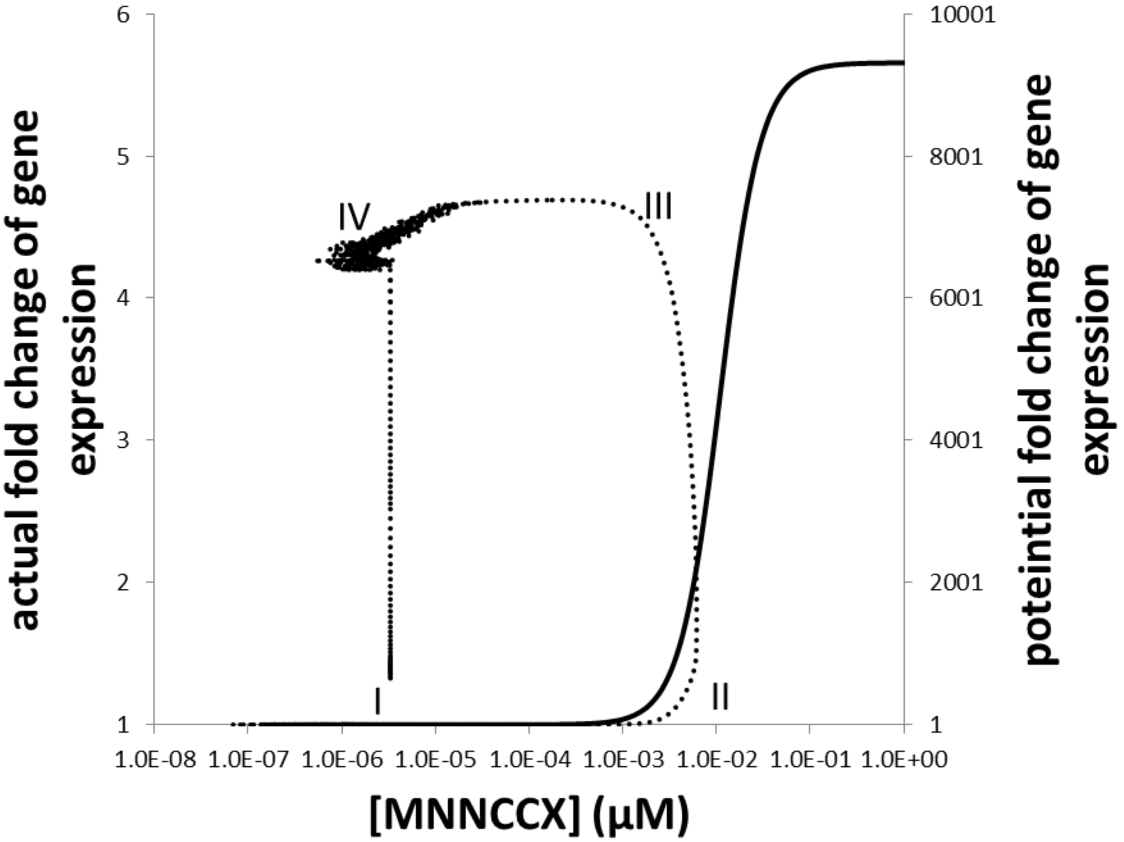

Fig. S16 Gene expression accumulates all information during the lifetime of the transient calcium signature (Fig. 3a) for binding affinity between the active complex $4Ca^{2+}$–CaM–CAMTA and DNA to be 1.1e-2 μM. Solid line (right *y*-axis): potential fold change of gene expression if the concentration of $4Ca^{2+}$–CaM–CAMTA stays at each concentration sufficiently long enough that a steady-state is established at each concentration. Dashed line (left *y*-axis): actual fold change of gene expression for transient calcium signature (Fig. 3a). Gene expression accumulates all information from the transient calcium signature (Fig. 3a) in a similar manner to the first cycle of Fig. 7 (points I–IV), as analysed in the main text.

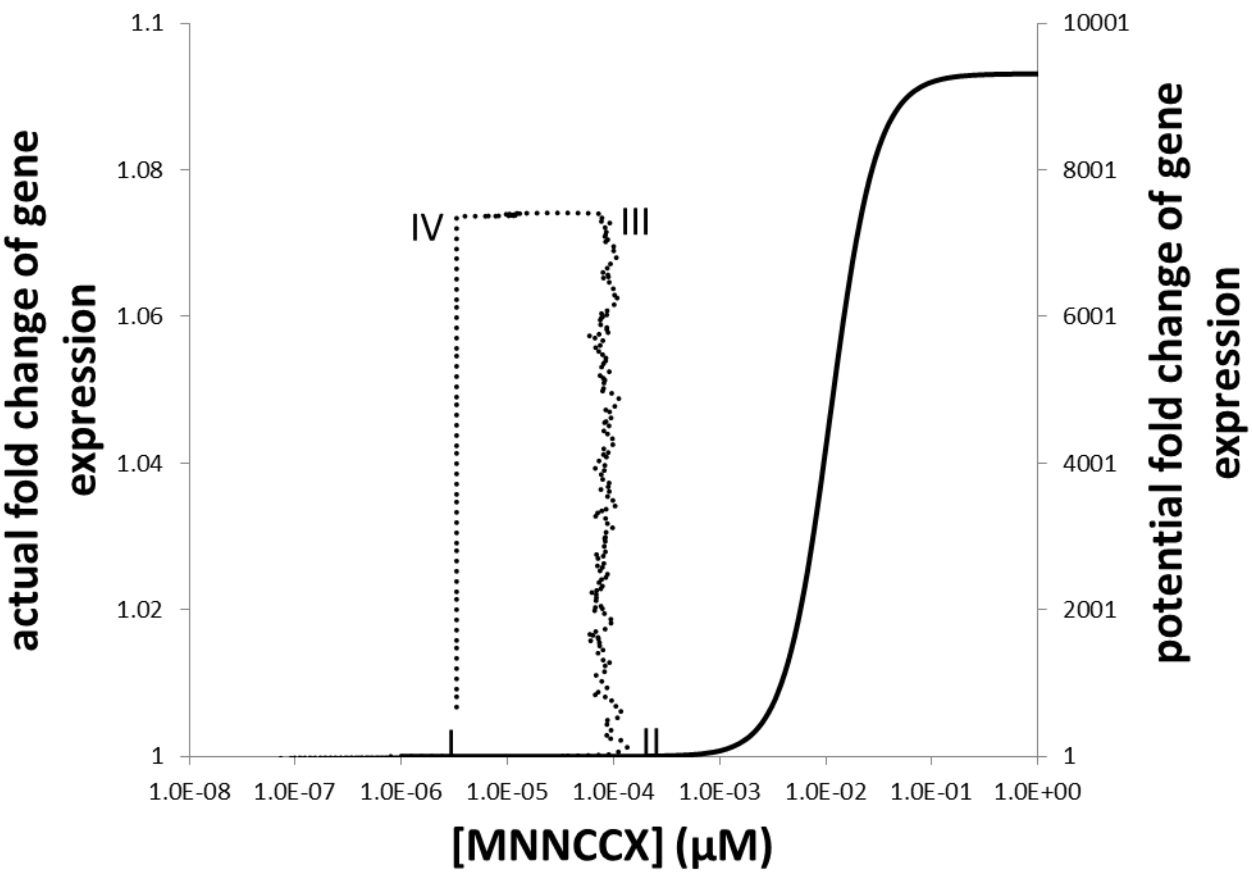

Fig. S17 Gene expression accumulates all information during the lifetime of the prolonged calcium signature (Fig. 4a) for binding affinity between the active complex $4Ca^{2+}$–CaM–CAMTA and DNA to be 1.1e-2 µM. Solid line (right *y*-axis): potential fold change of gene expression if the concentration of $4Ca^{2+}$–CaM–CAMTA stays at each concentration sufficiently long enough that a steady-state is established at each concentration. Dashed line (left *y*-axis): actual fold change of gene expression for prolonged calcium signature (Fig. 4a). Gene expression accumulates all information from the prolonged calcium signature (Fig. 4a) in a similar manner to the first cycle of Fig. 7 (points I–IV), as analysed in the main text.

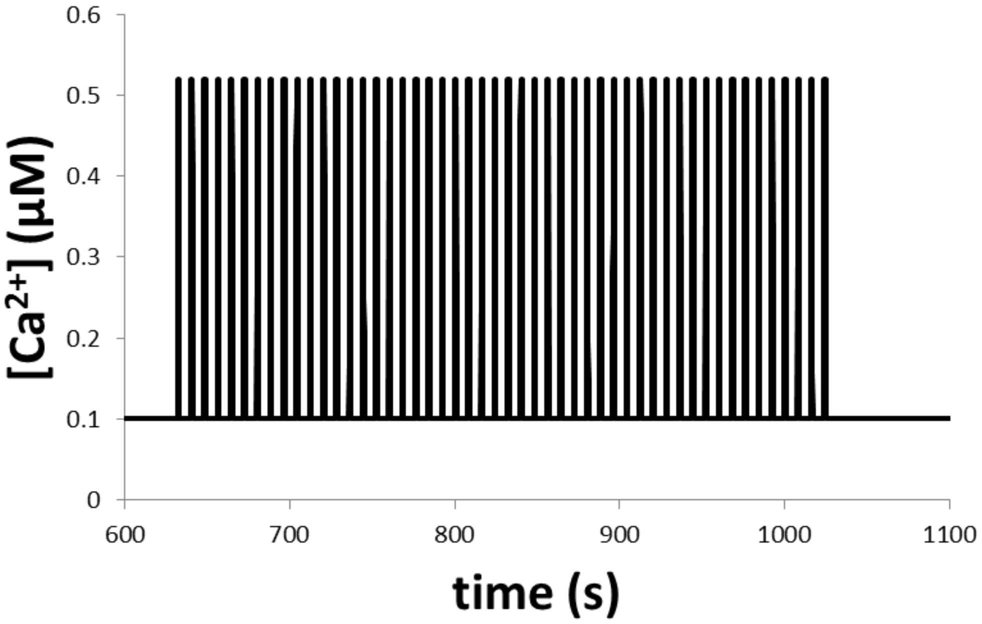

Fig. S18 The reconstructed piecewise calcium signature with T=8 s and all other parameters are exactly the same as in Fig. 8(a) (A= 0.16µM, $[Ca^{2+}]_{max}$ =0.52 µM and $[Ca^{2+}]_{min}$ =0.10 µM).

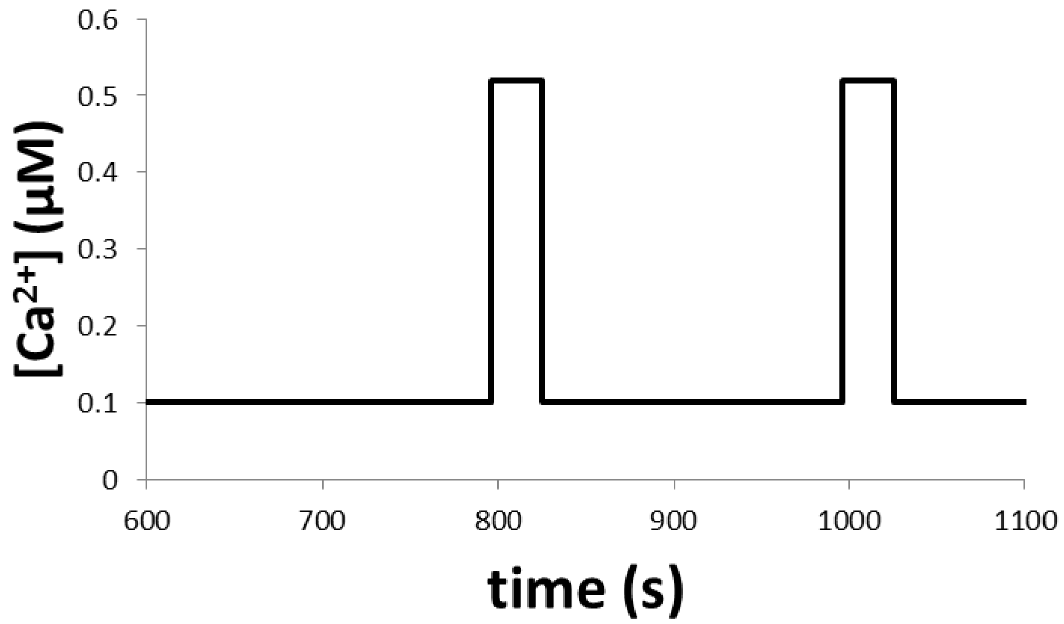

Fig. S19 The reconstructed piecewise calcium signature with T=200 s and all other parameters are exactly the same as in Fig. 8(a) (A= 0.16µM, $[Ca^{2+}]_{max}$ =0.52 µM and $[Ca^{2+}]_{min}$ =0.10 µM).

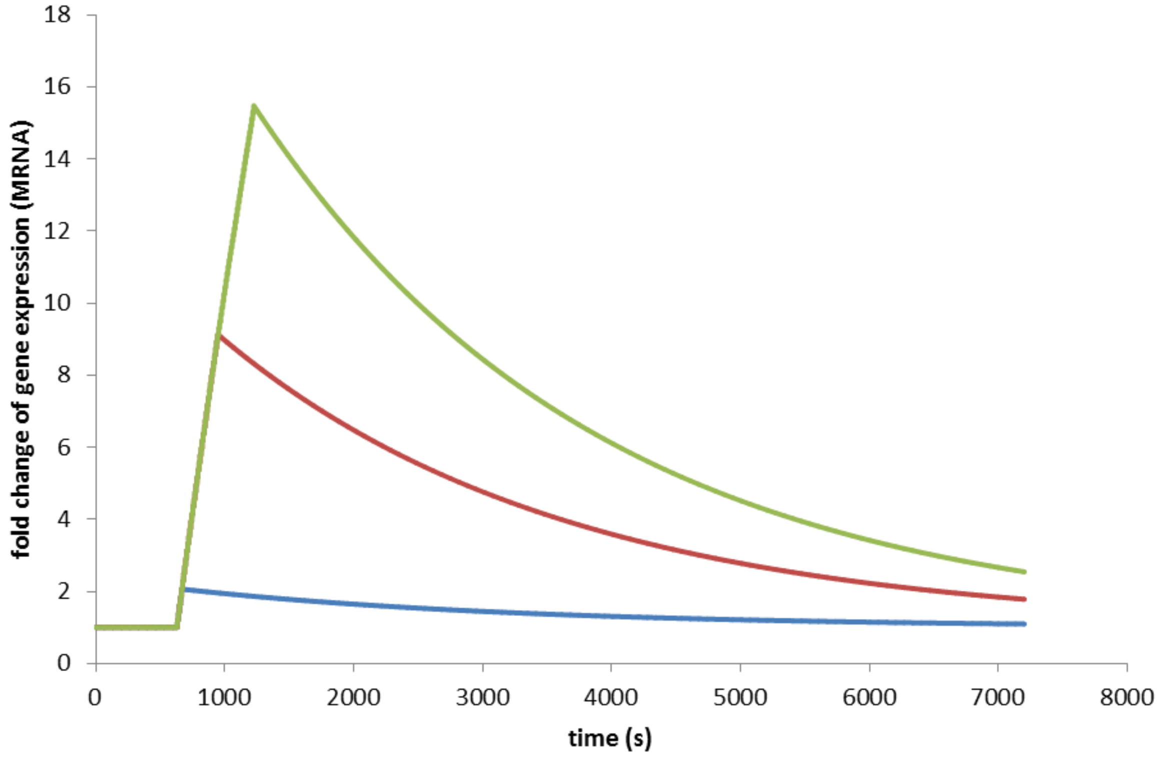

Fig. S20 Effects of the number of calcium spikes on fold change in gene expression induced by three piecewise calcium signatures that are reconstructed using the oscillatory calcium signature (Fig. 2a). Binding affinity (K_d) between the active complex $4Ca^{2+}$–CaM–CAMTA and DNAis 1.1e-2 μM. A= 0.16μM, $[Ca^{2+}]_{max}$ =0.52 μM and $[Ca^{2+}]_{min}$ =0.10 μM, T=8 s (a) blue line: 5 calcium spikes (i.e. the duration is 40 s); (b) red line: 40 calcium spikes (i.e. the duration is 320 s); (c) green line: 75 calcium spikes (i.e. the duration is 600 s).

Notes S1 Modelling equations

1. Modelling equations for the binding of calcium, CaM and CAMTA

$$\frac{d[M_{-----}]}{dt} = -k_{on(R1)}[M_{-----}][Ca^{2+}] + k_{off(R1)}[M_{--C--}] - k_{on(R3)}[M_{-----}][Ca^{2+}] + k_{off(R3)}[M_{N----}] - k_{on(R15)}[M_{-----}][X] + k_{off(R15)}[M_{----X}]$$

$$\frac{d[M_{--C--}]}{dt} = k_{on(R1)}[M_{-----}][Ca^{2+}] - k_{off(R1)}[M_{--C--}] - k_{on(R2)}[M_{--C--}][Ca^{2+}] + k_{off(R2)}[M_{--CC-}] - k_{on(R5)}[M_{--C--}][Ca^{2+}] + k_{off(R5)}[M_{N-C--}] - k_{on(R18)}[M_{--C--}][X] + k_{off(R18)}[M_{--C-X}]$$

$$\frac{d[M_{--CC-}]}{dt} = k_{on(R2)}[M_{--C--}][Ca^{2+}] - k_{off(R2)}[M_{--CC-}] - k_{on(R7)}[M_{--CC-}][Ca^{2+}] + k_{off(R7)}[M_{N-CC-}] - k_{on(R20)}[M_{--CC-}][X] + k_{off(R20)}[M_{--CCX}]$$

$$\frac{d[M_{N----}]}{dt} = k_{on(R3)}[M_{-----}][Ca^{2+}] - k_{off(R3)}[M_{N----}] - k_{on(R4)}[M_{N----}][Ca^{2+}] + k_{off(R4)}[M_{NN---}] - k_{on(R9)}[M_{N----}][Ca^{2+}] + k_{off(R9)}[M_{N-C--}] - k_{on(R14)}[M_{N----}][X] + k_{off(R14)}[M_{N---X}]$$

$$\frac{d[M_{N-C--}]}{dt} = k_{on(R5)}[M_{--C--}][Ca^{2+}] - k_{off(R5)}[M_{N-C--}] - k_{on(R6)}[M_{N-C--}][Ca^{2+}] + k_{off(R6)}[M_{NNC--}] + k_{on(R9)}[M_{N----}][Ca^{2+}] - k_{off(R9)}[M_{N-C--}] - k_{on(R10)}[M_{N-C--}][Ca^{2+}] + k_{off(R10)}[M_{N-CC-}] - k_{on(R17)}[M_{N-C--}][X] + k_{off(R17)}[M_{N-C-X}]$$

$$\frac{d[M_{N-CC-}]}{dt} = k_{on(R7)}[M_{--CC-}][Ca^{2+}] - k_{off(R7)}[M_{N-CC-}] - k_{on(R8)}[M_{N-CC-}][Ca^{2+}] + k_{off(R8)}[M_{NNCC-}] + k_{on(R10)}[M_{N-C--}][Ca^{2+}] - k_{off(R10)}[M_{N-CC-}] - k_{on(R19)}[M_{N-CC-}][X] + k_{off(R19)}[M_{N-CCX}]$$

$$\frac{d[M_{NN---}]}{dt} = k_{on(R4)}[M_{N----}][Ca^{2+}] - k_{off(R4)}[M_{NN---}] - k_{on(R11)}[M_{NN---}][Ca^{2+}] + k_{off(R11)}[M_{NNC--}] - k_{on(R13)}[M_{NN---}][X] + k_{off(R13)}[M_{NN--X}]$$

$$\frac{d[M_{NNC--}]}{dt} = k_{on(R6)}[M_{N-C--}][Ca^{2+}] - k_{off(R6)}[M_{NNC--}] + k_{on(R11)}[M_{NN---}][Ca^{2+}] - k_{off(R11)}[M_{NNC--}] - k_{on(R12)}[M_{NNC--}][Ca^{2+}] + k_{off(R12)}[M_{NNCC-}] - k_{on(R16)}[M_{NNC--}][X] + k_{off(R16)}[M_{NNC-X}]$$

$$\frac{d[M_{NNCC-}]}{dt} = k_{on(R8)}[M_{N-CC-}][Ca^{2+}] + k_{off(R8)}[M_{NNCC-}] + k_{on(R12)}[M_{NNC--}][Ca^{2+}] - k_{off(R12)}[M_{NNCC-}] - k_{on(R33)}[M_{NNCC-}][X] + k_{off(R33)}[M_{NNCCX}]$$

$$\frac{d[M_{----X}]}{dt} = -k_{on(R21)}[M_{----X}][Ca^{2+}] + k_{off(R21)}[M_{--C-X}] - k_{on(R23)}[M_{----X}][Ca^{2+}] + k_{off(R23)}[M_{N---X}] + k_{on(R15)}[M_{-----}][X] - k_{off(R15)}[M_{----X}]$$

$$\frac{d[M_{--C-X}]}{dt} = k_{on(R21)}[M_{----X}][Ca^{2+}] - k_{off(R21)}[M_{--C-X}] - k_{on(R22)}[M_{--C-X}][Ca^{2+}] + k_{off(R22)}[M_{--CCX}] - k_{on(R25)}[M_{--C-X}][Ca^{2+}] + k_{off(R25)}[M_{N-C-X}] + k_{on(R18)}[M_{--C--}][X] - k_{off(R18)}[M_{--C-X}]$$

$$\frac{d[M_{--CCX}]}{dt} = k_{on(R22)}[M_{--C-X}][Ca^{2+}] - k_{off(R22)}[M_{--CCX}] - k_{on(R27)}[M_{--CCX}][Ca^{2+}] + k_{off(R27)}[M_{N-CCX}] + k_{on(R20)}[M_{--CC-}][X] - k_{off(R20)}[M_{--CCX}]$$

$$\frac{d[M_{N---X}]}{dt} = k_{on(R23)}[M_{----X}][Ca^{2+}] - k_{off(R23)}[M_{N---X}] - k_{on(R24)}[M_{N---X}][Ca^{2+}] + k_{off(R24)}[M_{NN--X}] - k_{on(R29)}[M_{N---X}][Ca^{2+}] + k_{off(R29)}[M_{N-C-X}] + k_{on(R14)}[M_{N----}][X] - k_{off(R14)}[M_{N---X}]$$

$$\frac{d[M_{N-C-X}]}{dt} = k_{on(R25)}[M_{--C-X}][Ca^{2+}] - k_{off(R25)}[M_{N-C-X}] - k_{on(R26)}[M_{N-C-X}][Ca^{2+}] + k_{off(R26)}[M_{NNC-X}] + k_{on(R29)}[M_{N---X}][Ca^{2+}] - k_{off(R29)}[M_{N-C-X}] - k_{on(R30)}[M_{N-C-X}][Ca^{2+}] + k_{off(R30)}[M_{N-CCX}] + k_{on(R17)}[M_{N-C--}][X] - k_{off(R17)}[M_{N-C-X}]$$

$$\frac{d[M_{N-CCX}]}{dt} = k_{on(R27)}[M_{--CCX}][Ca^{2+}] - k_{off(R27)}[M_{N-CCX}] - k_{on(R28)}[M_{N-CCX}][Ca^{2+}] + k_{off(R28)}[M_{NNCCX}] + k_{on(R30)}[M_{N-C-X}][Ca^{2+}] - k_{off(R30)}[M_{N-CCX}] + k_{on(R19)}[M_{N-CC-}][X] - k_{off(R19)}[M_{N-CCX}]$$

$$\frac{d[M_{NN--X}]}{dt}=k_{on(R24)}[M_{N---X}][Ca^{2+}]-k_{off(R24)}[M_{NN--X}]-k_{on(R31)}[M_{NN--X}][Ca^{2+}]+k_{off(R31)}[M_{NNC-X}]$$
$$+k_{on(R13)}[M_{NN---}][X]-k_{off(R13)}[M_{NN--X}]$$

$$\frac{d[M_{NNC-X}]}{dt}=k_{on(R26)}[M_{N-C-X}][Ca^{2+}]-k_{off(R26)}[M_{NNC-X}]+k_{on(R31)}[M_{NN--X}][Ca^{2+}]-k_{off(R31)}[M_{NNC-X}]$$
$$-k_{on(R32)}[M_{NNC-X}][Ca^{2+}]+k_{off(R32)}[M_{NNCCX}]+k_{on(R16)}[M_{NNC--}][X]-k_{off(R16)}[M_{NNC-X}]$$

$$\frac{d[M_{NNCCX}]}{dt}=k_{on(R28)}[M_{N-CCX}][Ca^{2+}]-k_{off(R28)}[M_{NNCCX}]+k_{on(R32)}[M_{NNC-X}][Ca^{2+}]-k_{off(R32)}[M_{NNCCX}]$$
$$+k_{on(R33)}[M_{NNCC-}][X]-k_{off(R33)}[M_{NNCCX}]$$

CaM_t : the total concentration of CaM, which is the summation of free CaM and all CaM complexes.

$$CaM\_t=[M_{-----}]+[M_{--C--}]+[M_{--CC-}]+[M_{N----}]+[M_{N-C--}]+[M_{N-CC-}]$$
$$+[M_{NN---}]+[M_{NNC--}]+[M_{NNCC-}]+[M_{----X}]+[M_{--C-X}]+[M_{--CCX}]$$
$$+[M_{N---X}]+[M_{N-C-X}]+[M_{N-CCX}]+[M_{NN--X}]+[M_{NNC-X}]+[M_{NNCCX}]$$

X_t : the total concentration of CAMTA, which is the summation of free CAMTA and all CAMTA complexes.

$$X\_t=[X]+[M_{----X}]+[M_{--C-X}]+[M_{--CCX}]+[M_{N---X}]+[M_{N-C-X}]$$
$$+[M_{N-CCX}]+[M_{NN--X}]+[M_{NNC-X}]+[M_{NNCCX}]$$

2. Modelling equations for gene expression

$$\frac{d[mRNA]}{at}=k_1+\frac{k_2\left(\frac{[MNNCCX]}{k_4}\right)^n}{1+\left(\frac{[MNNCCX]}{k_4}\right)^n}-k_3[mRNA]$$

Where k_1 is the base rate for gene transcription, k_2 is the maximal rate for CAMTA-regulated gene transcription, k_3 is the decay rate constant for the mRNA, k_4 is the binding affinity between $4Ca^{2+}$-CaM-CAMTA complex and DNA, n is Hill coefficient.
